# Genome-scale data resolve ancestral rock-inhabiting lifestyle in Dothideomycetes (Ascomycota)

**DOI:** 10.1186/s43008-019-0018-2

**Published:** 2019-10-30

**Authors:** Claudio G. Ametrano, Felix Grewe, Pedro W. Crous, Stephen B. Goodwin, Chen Liang, Laura Selbmann, H. Thorsten Lumbsch, Steven D. Leavitt, Lucia Muggia

**Affiliations:** 1 Department of Life Sciences, University of Trieste, via Giorgieri 10, 34127 Trieste, Italy; 2 Grainger Bioinformatics Center and Integrative Research Center, Science and Education, Field Museum of Natural History, 1400 S Lake Shore Drive, Chicago, IL 60605 USA; 3 Westerdijk Fungal Biodiversity Institute, P.O. Box 85176, 3508 AD Utrecht, The Netherlands; 4 USDA-ARS, Crop Production and Pest Control Research Unit and Department of Botany and Plant Pathology, Purdue University, 915 West State Street, West Lafayette, IN 47907-2054 USA; 5 College of Plant Health and Medicine, Qingdao Agricultural University, Qingdao, 266109 China; 6 Department of Ecological and Biological Sciences, University of Tuscia, Largo dell’ Università, 01100 Viterbo, Italy; 7 Italian National Antarctic Museum (MNA), Mycological Section, Genoa, Italy; 8 Department of Biology and M.L. Bean Life Science Museum, Brigham Young University, 4102 Life Science Building, Provo, UT 84602 USA

**Keywords:** *Lichenothelia*, Phylogenomics, *Saxomyces*, Species tree, Supermatrix, Supertree

## Abstract

Dothideomycetes is the most diverse fungal class in Ascomycota and includes species with a wide range of lifestyles. Previous multilocus studies have investigated the taxonomic and evolutionary relationships of these taxa but often failed to resolve early diverging nodes and frequently generated inconsistent placements of some clades. Here, we use a phylogenomic approach to resolve relationships in Dothideomycetes, focusing on two genera of melanized, extremotolerant rock-inhabiting fungi, *Lichenothelia* and *Saxomyces*, that have been suggested to be early diverging lineages. We assembled phylogenomic datasets from newly sequenced (4) and previously available genomes (238) of 242 taxa. We explored the influence of tree inference methods, supermatrix vs. coalescent-based species tree, and the impact of varying amounts of genomic data. Overall, our phylogenetic reconstructions provide consistent and well-supported topologies for Dothideomycetes, recovering *Lichenothelia* and *Saxomyces* among the earliest diverging lineages in the class. In addition, many of the major lineages within Dothideomycetes are recovered as monophyletic, and the phylogenomic approach implemented strongly supports their relationships. Ancestral character state reconstruction suggest that the rock-inhabiting lifestyle is ancestral within the class.

## INTRODUCTION

Dothideomycetes is the largest and most diverse fungal class of ascomycetes, comprised of c. 20,000 species (Jaklitsch et al. 2016) classified into 105 families (Hyde et al. 2013) and 32 orders (Liu et al. 2017). The class encompasses great variation of fungal lifestyles, including saprotrophs, plant pathogens, endophytes, epiphytes, fungicolous, lichenized, lichenicolous, and free-living rock-inhabiting fungi.

Several phylogenetic inferences have been generated to resolve taxonomy and evolutionary relationships within Dothideomycetes at different systematic levels (e.g. Schoch et al. 2006, Schoch et al. 2009; Nelsen et al. 2009; Ruibal et al. 2009; Hyde et al. 2013; Muggia et al. 2015; Liu et al. 2017; Ametrano et al. 2019). These analyses usually considered wide taxon sampling and were based on combinations of nuclear, mitochondrial, and protein-coding loci. However, previous studies inferred inconsistent placement of some orders or families, particularly among the basal nodes. In spite of the increased use of genome-scale data to resolve long-standing evolutionary and taxonomic issues (Chan and Ragan 2013), phylogenomic approaches are rather uncommon for Dothideomycetes, although about 250 sequenced genomes of its representatives have been sequenced. Within this class, genome sequencing efforts have largely focused on plant and human pathogenic fungi (Hane et al. 2007; Ohm et al. 2012; Raffaele and Kamoun^2012^), and fungi with a certain ecological (e.g., melanized, halotolerant yeast; Gostinčar et al. 2011) or economical interest (e.g. carbohydrate degraders; Prenafeta-Boldu et al. 2006; Sterflinger 2006; Nai et al. 2013). On the other hand, the most inconspicuous taxa have been largely neglected in genomic research – especially those belonging to the group of melanized, meristematic, rock-inhabiting fungi (RIF). Only recently few Antarctic RIF genomes have become available (Coleine et al. 2017). This shortcoming is likely due to the difficulty to retrieve those species in nature, isolate them axenically in vitro and their extremely slow growth rate in culture.

Two dothidealean genera, *Lichenothelia* and *Saxomyces*, are iconic representatives of RIF (Muggia et al. 2015; Selbmann et al. 2014; Ametrano et al. 2019). Species of *Lichenothelia* and *Saxomyces* are widespread worldwide, occurring on exposed rocks, often in extreme environments, and having evolved lifestyles on nutrient-poor substrates. Because they can survive in harsh environments characterized by high solar radiation, very high and very low temperatures, and drought stress, they have been recognized within the group of polyextremotolerant fungi (Gostinčar et al. 2012). *Lichenothelia* species are of particular interest because they exhibit a multiplicity of lifestyles, e.g. non-lichenized rock-inhabiting, parasitic on lichens, and loosely associated with green algae on rocks. Due to its affinity towards algae, *Lichenothelia* has been historically considered an evolutionary link between the non-lichenized Dothideomycetes and the lichenized Lecanoromycetes (Hawksworth 1981; Muggia et al. 2013). Recent phylogenetic analyses have identified *Lichenothelia* and *Saxomyces* as two individually monophyletic lineages, but their phylogenetic placement within Dothideomycetes remained unresolved (Ametrano et al. 2019). Therefore, more information from *Lichenothelia* and *Saxomyces* genomes is required to better understand their genetic diversity and evolutionary relationships with other closely related dothideomycetous taxa with varying lifestyles.

Here, we present a phylogenomic study on the evolutionary relationships of *Lichenothelia* and *Saxomyces* within Dothideomycetes. Genome-scale data from de novo genome assemblies of two species of *Lichenothelia* and two of *Saxomyces* were added to a supermatrix including genes of most Dothideomycetes taxa for which whole-genome data were available. Our study aimed to (i) generate a genome-scale phylogeny of Dothideomycetes to resolve the phylogenetic placement of still unsupported lineages and in particular clarify that of *Lichenothelia* and *Saxomyces* and their relationships with other RIF lineages within the class, (ii) assess whether and to which extent the amount of genetic information, the alignment processing and the phylogenomic reconstruction method impact the final phylogenetic inference, and (iii) assess the minimum amount of genomic information needed to generate a topology that agrees with the phylogeny generated with the entire set of genes.

## MATERIALS AND METHODS

### Cultured strains, DNA extraction and sequencing

Fungal strains representing *Lichenothelia* and *Saxomyces* species were available from previous culture isolations reported by Muggia et al. (2013, 2015), Selbmann et al. (2014) and Ametrano et al. (2017, 2019). The strains for genome sequencing are: *Lichenothelia convexa* L1844 (LMCC0061, MUT5682); *Lichenothelia intermixta* L2282 (LMCC0543); *Saxomyces alpinus* CCFEE5470 (CBS135222); and *Saxomyces americanus* L1853 (LMCC0060, MUT5853). Strains were sub-cultured on malt-yeast medium (MY, Ahmadjian 1967) at 20 °C, and DNA was extracted as soon as the mycelia grew to a sufficient biomass (after about 4 weeks).

The fungal biomass was removed from the growth medium, ground in liquid nitrogen and genomic DNA was extracted using the ZR Fungal/Bacterial DNA MicroPrep™ Kit (Zymo Research) according to the manufacturer’s protocol. The quality of the genomic DNA was checked by gel electrophoresis on 0.8% agarose gel, which showed a sharp genomic DNA band with a small amount of degraded nucleic acid. DNA concentration was 7–11 ng/μl (175–275 ng of DNA), and the nucLSU rDNA was successfully sequenced to confirm the identity of the strains (100% sequence identity). The four genomic DNA extractions were sent to the University of Illinois at Chicago sequencing facility for library preparation (Nextera XT) and sequencing on an Illumina MiSeq platform. The strain of *Lichenothelia convexa* was sequenced with a coverage three times deeper than the other three strains to obtain a better assembly.

### Bioinformatics

**Fig. 1:**
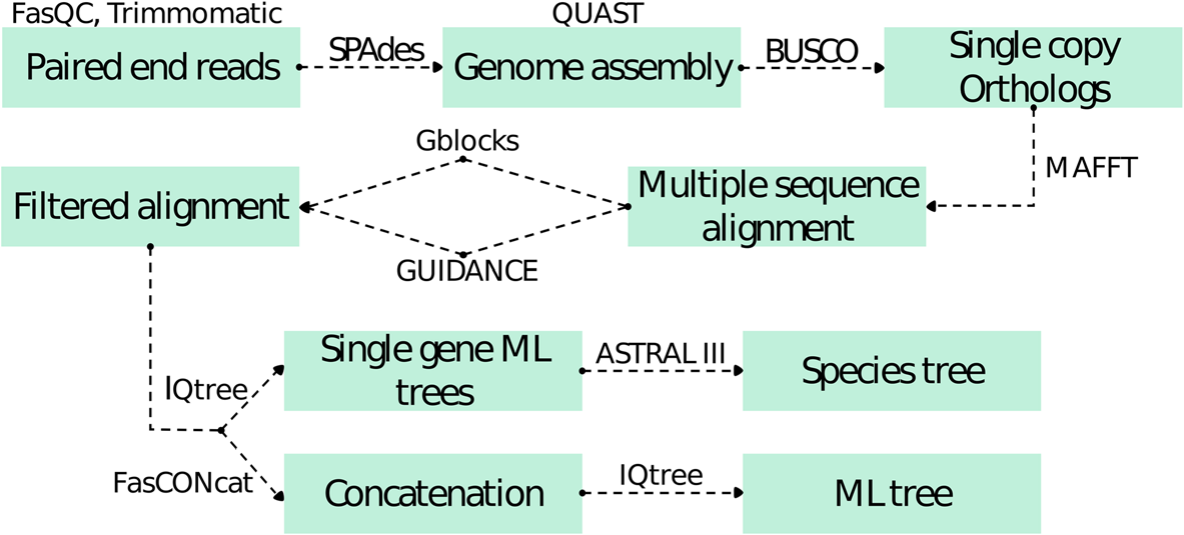
Flow chart reporting the bioinformatic pipeline used for the analyses. Input and output of the pipeline are reported inside the boxes, the software used for each pipeline step is reported above or between the boxes

A bioinformatic pipeline consisting of several programs was generated to extract single-copy genes from whole-genome assemblies and create individual gene alignments and phylogenies (Fig. 1). Fastq files containing 2 × 150 bp paired-end (PE) reads were quality filtered with Trimmomatic 0.35 (Bolger et al. 2014) to remove sequencing adapters, low-quality nucleotides and short reads. We changed the recommended settings to LEADING:10, TRAILING:10, and MINLEN:25 to trim the ends of the reads when the quality was below 10 and subsequently remove sequences shorter than 25 bases. A quality check was performed with FastQC 0.11.5 both before and after reads trimming. High quality, paired-end and orphan reads were then assembled with SPAdes 3.5.0 using default k-mer lengths based on read length (kmer of 21, 33 and 55 bp for 151 bp reads) (Bankevich et al. 2012). The assemblies from the multi k-mer SPAdes approach were checked with Quality Assessments Tool (QUAST 4.5, Gurevich et al. 2013). The assembly completeness was also analyzed with Benchmarking Universal Single Copy Orthologs (BUSCO 3.0.1; Waterhouse et al. 2017). In addition, 238 whole-genome assemblies of other Dothideomycetes were downloaded from NCBI GenBank and the JGI Genome portal (Additional file 1: Table S1; Galagan et al. 2005; Fedorova et al. 2008; Sharpton et al. 2009; Ellwood et al. 2010; Desjardins et al. 2011; Goodwin et al. 2011; Rouxel et al. 2011; Stukenbrock et al. 2011; Chan et al. 2012; Hu et al. 2012; Joardar et al. 2012; Ng et al. 2012; Ohm et al. 2012; Spatafora et al. 2012; Blanco-Ulate et al. 2013; Condon et al. 2013; Lenassi et al. 2013; Yew et al. 2013; Aragona et al. 2014; Bihon et al. 2014; Chan et al. 2014; Cooke et al. 2014; Gao et al. 2014; Gostinčar et al., 2014; Han et al. 2014; Soliai et al. 2014; Sterflinger et al. 2014; van der Nest et al. 2014; Yang et al. 2014; Franco et al. 2015; Grandaubert et al. 2015; Kuan et al. 2015; Morales-Cruz et al. 2015; Orner et al. 2015; Shaw et al. 2015; Shiller et al. 2015; Vaghefi et al. 2015; Wingfield et al. 2015; Bock et al. 2016; Chang et al. 2016; Mosier et al. 2016; Nguyen et al. 2016; Peter et al. 2016; Verma et al. 2016; Wang et al. 2016; Xu et al. 2016; Zeiner et al. 2016; Coleine et al. 2017; Marsberg et al. 2017; Mondo et al. 2017; Shrestha et al. 2017; Teixeira et al. 2017; Zeng et al. 2017; Knapp et al. 2018; Lopez et al. 2018) and processed with BUSCO. All assemblies of Dothideomycetes available during June 2017, when the dataset was built, were included, except those which were taxonomically mis-assigned during test runs of the dataset. Distribution of the BUSCO completeness of the assemblies was assessed for outliers with Thompson Tau test (Thompson 1935). BUSCO evaluation of the completeness of the genome assemblies is based on a set of orthologous genes (OrthoDB; Zdobnov et al. 2016) present in the members of the taxonomic group of interest, Pezizomycotina ortholog gene set was used for Dothideomycetes. Among the genes predicted by BUSCO, only single-copy orthologs, which are suitable for phylogenetic inference, were selected and used for subsequent analyses. Orthologous genes which were present in single copy but predicted in multiple possible versions were also discarded. Selected single-copy genes from each taxon were aligned with MAFFT 7 (Katoh and Standley 2013) using default parameters. As the alignment filtering method can affect the output of the subsequent phylogenetic inferences (Tan et al. 2015), sometimes worsening the phylogenetic results, sequence alignments from MAFFT were filtered either with Gblocks (Castresana 2000) or with GUIDe tree-based AligNment ConfidencE (GUIDANCE 2.02, Penn et al. 2010).

### Phylogenomic analyses

The phylogenetic reliability of the generated dataset was tested with different numbers of genes, alignment filtering methods and tree reconstruction methods. Five individual datasets were constructed: (i) a dataset of genes longer than 1000 bp after Gblocks trimming (“> 1 kb Gblocks” dataset) and (ii) one after GUIDANCE trimming with less than 50% of gaps (“> 1 kb GUIDANCE” dataset); (iii) the complete set of retrieved genes including both genes longer and shorter than 1 kb after Gblocks filtering (“Complete Gblocks” dataset) and (iv) GUIDANCE filtering (“Complete GUIDANCE” dataset); (v) a dataset without any missing data for estimating the impact of missing data on the tree, hence reduced both in gene number and taxa (“No missing” dataset). The individual gene alignments of each dataset were either used for individual gene tree calculations, or concatenated into a supermatrix with FasCONcat 1.0 (Kück and Meusemann 2010) (Fig. 1). Maximum likelihood (ML) phylogenetic inferences from the supermatrix, as well as the single-locus inferences, were produced with IQTree 1.6.1 (Nguyen et al. 2014) using 1000 replicates of ultra-fast bootstrap (−bb) to get node support values (Hoang et al. 2017) and Model Finder Plus (−MFP) to select the most suitable nucleotide substitution model. Gene trees resulting from single-locus inferences were further combined in a supertree with the coalescent-based species tree estimation software ASTRAL III (Zhang et al. 2017). The resulting topologies were compared with normalized Robinson-Foulds distance (RF, Robinson and Foulds 1981).

As the analysis of a genome-based supermatrix with bootstrap support can be highly computationally demanding, an alternative, customized, resampling strategy was tested in the analyses on the “> 1 kb Gblocks” concatenated alignment. Thirty runs of IQTree were carried out on reduced concatenated matrices made of an increasing, randomly selected number of columns from this alignment. The phylogeny resulting from the complete supermatrix was taken as reference and used to compute RF distances in RAxML 8.2 (Stamatakis 2014) with the phylogenies generated from the re-sampled alignments. Resampling was performed without replacement and the sampling effort was increased until no statistically significant difference among RF distance distributions was detected (one-way ANalysis Of Variance (ANOVA) *p* < 0.01 and post hoc pairwise tests: Tukey, Bonferroni and Scheffe, Statistica 6).

### Ancestral character state reconstruction

Ancestral character state reconstruction analyses were carried out based on the phylogeny produced by the concatenation of genes from dataset (i). Both Maximum Parsimony and Maximum Likelihood approaches were tested using “Trace character History” option in Ancestral State Reconstruction package of MESQUITE 3.6 (Maddison & Maddison 2018). As the number of states for the character “lifestyle” is large (10; Additional file 5: Table S5) a One-parameter Markov k-state model (Mk1; Lewis 2001) was used; it is a generalization of the Jukes-Cantor model.

## RESULTS

### Assembly statistics and completeness of the genomes

After quality filtering, total numbers of PE reads for each species were 39.4 million for *Lichenothelia convexa* L1844, 9.7 million for *L. intermixta* L2282, 8.6 million for *Saxomyces alpinus* CCFEE 5470 and 9.9 million for *S. americanus* L1853. Assembly statistics are reported in Table 1. BUSCO assembly completeness analysis on 3156 orthologous genes for the subphylum Pezizomycotina recovered 93.1% for *L. convexa*, 92.3% for *L. intermixta*, 46.1% for *S. alpinus* and 95.7% for *S. americanus*. The mean and standard deviation for the entire assembly dataset of the 242 Dothideomycetes is 96.3 ± 6 (the complete output of the BUSCO analysis is reported in Additional file 6: Figure S1).

**Table 1:** Summary information for sequencing and assembly of the genomes from four species in the genera *Lichenothelia* and *Saxomyces*

Sample	Reads (M)	Contings (> 500 bp)	Largest contig (bp)	N50 (bp)	L50	Assembly size (Mbp)
*Lichenothelia convexa* L1844	39.4	1669	539,045	61,89	165	36.6
*Lichenothelia intermixta* L2282	9.7	3619	205,447	35,632	233	29.4
*Saxomyces alpinus* CCFEE 5470	8.6	22,101	67,075	3086	3727	51.4
*Saxomyces americanus* L1853	9.9	10,111	115,917	18,029	561	42.3

The states estimated to be the best according to the Likelihood threshold are marked by an asterisk (*)

### Phylogenomic datasets

The “>1Kb Gblocks” dataset comprises 242 samples (including the four newly sequenced species of *Lichenothelia* and *Saxomyces*) and 664 genes longer than 1000 bp after Gblocks trimming. The total alignment length is 1.1 Mb. The “Complete Gblocks” dataset comprises the same samples, but it includes all the 2998 genes which are single copy, not predicted in multiple version and not empty after Gblocks trimming. As Gblocks does not allow gaps and it selects only perfectly aligned regions, many genes were drastically shortened. Hence the final length of the alignment is only twice as long as (2.2 Mb) the “>1Kb Gblocks” dataset, which is a subset of the total. The presence of samples characterized by relevant events of gene duplication or low-quality assemblies (Additional file 6: Figure S1) hampered finding genes among BUSCO orthologs, which were in common among all samples. The number of samples was therefore reduced to 229 taxa in the “No missing” dataset, retaining all *Lichenothelia* and *Saxomyces* assemblies. A total of 63 genes and a 31 Kb alignment were used to run the phylogenetic inference. The complete “>1Kb GUIDANCE dataset” comprises all 242 samples and, as GUIDANCE tends to be less strict than Gblocks, 1260 genes longer than 1 Kb have been included in the final alignment with a length of 7.4 Mb (Additional file 9: Figure S4).

### Comparison of the inferred phylogenies

**Fig. 2:**
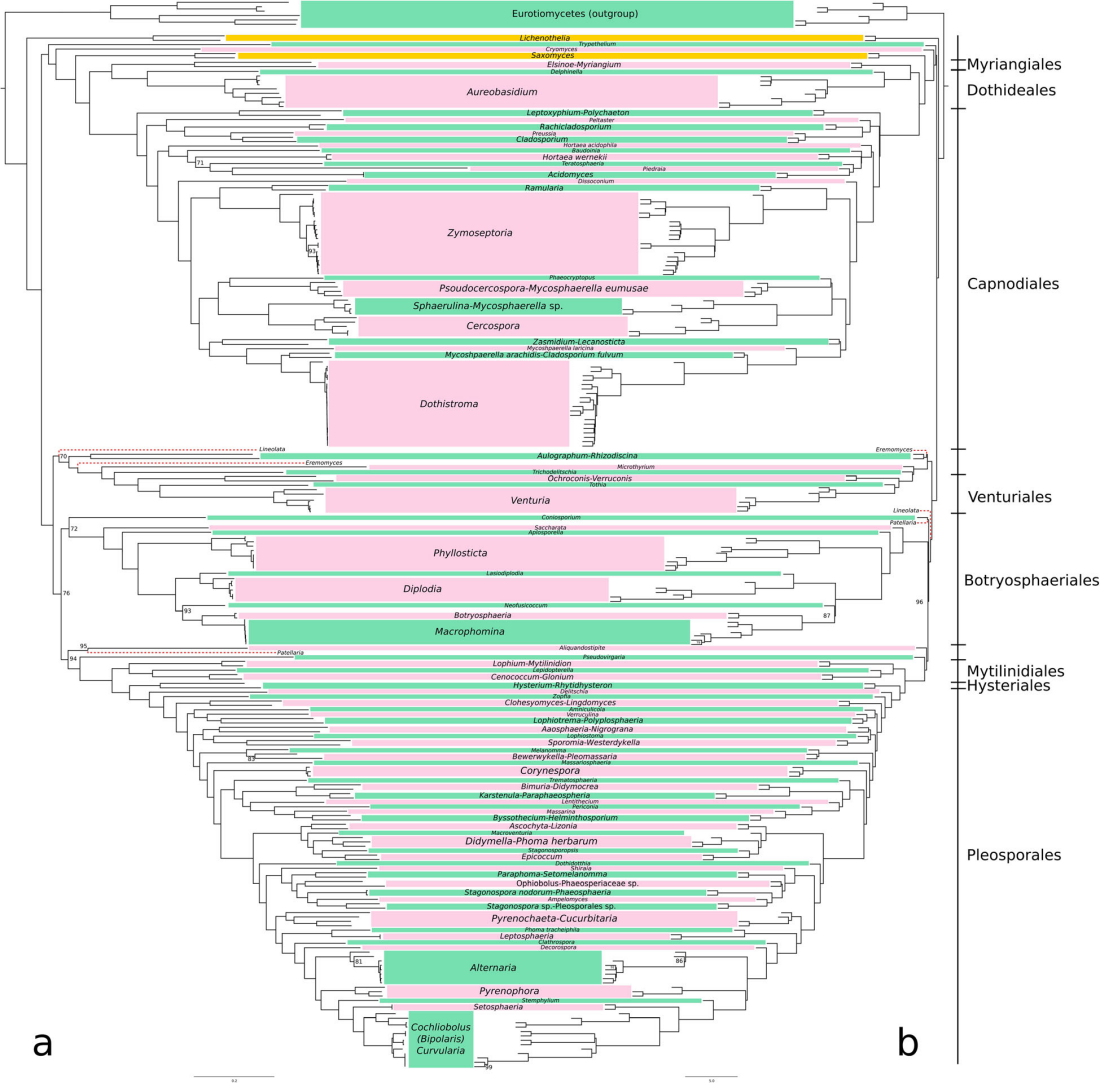
Phylogenomic inferences based on the concatenated supermatrix (**a**) and multispecies coalescent (**b**) approaches. The dataset was composed by 664 single-copy gene regions longer than 1 Kb (after alignment trimming with Gblocks). Topology mismatches between the phylogenies are highlighted by red dashed branch lines. Bootstrap support values lower than 100% are shown. *Lichenothelia* and *Saxomyces* clades are highlighted by orange boxes. Pink and green boxes alternatively delimit the other lineages, either represented by a single genus or by multiple genera

Phylogenies inferred from the same dataset but applying different reconstruction methods showed highly similar topologies. Only three incongruences were detected when comparing the two phylogenies obtained from the concatenation ML inference and the coalescent-based species tree inference of the “1Kb Gblocks” dataset, namely the placements of *Eremomyces bilateralis*, *Lineolata rhizophorae* and *Patellaria atrata* (Fig. 2a, b). The RF distance between these two phylogenies is indeed only 0.109, similar to the RF value obtained from the comparison of the two phylogenetic inferences based on the “1Kb GUIDANCE” dataset (0.100) (Additional file 2: Table S2 and Additional file 3: Table S3).

Changing both the starting dataset, (“Complete Gblocks” or “1Kb Gblocks”) and the reconstruction method (concatenation or coalescent-based) generated the most diverse topologies (RF distance value of 0.117; Additional file 2: Table S2). The most similar topologies were produced by the coalescent-based approach on “1Kb Gblocks” and “1Kb GUIDANCE” dataset (Additional file 10: Figure S5). Although these inferences are based on a rather different datasets of genes, they produced nearly identical topologies with a RF distance of 0.025 (Additional file 2: Table S2). This is remarkable, because the datasets were comprised of 664 and 1260 genes, respectively. Even if the same markers were considered in both datasets, the retained parts of the alignment are not the same, as they have been obtained using two different filtering methods. Only *Eremomyces bilateralis* had a different placement, though unsupported (ultrafast bootstrap value lower than 95).

Three runs of the “1Kb Gblocks” dataset with the concatenation approach produced perfectly congruent topologies which only differs in the support values of some of the less-supported lineages, as shown by weighed RF distance values, which are very close to zero. Few other taxa show an unstable position within the phylogeny. *Neofusicoccum parvum*, for instance, is basal to the *Botryosphaeria-Macrophomina* clade using the “1Kb Gblocks” dataset, while it is basal to the *Lasidiplodia-Diplodia-Botryosphaeria-Macrophomina* clade considering the “Complete Gblocks” dataset run as a concatenated supermatrix (Additional file 7: Figure S2). However, these phylogenetic positions are not fully supported by the ultrafast bootstrap value.

The “No missing” dataset, although built on both a reduced number of samples (229) and markers (63), produced highly similar results when considering the phylogeny obtained from the concatenation (Additional file 8: Figure S3a). However, when the same dataset is analyzed with the coalescent-based approach (Additional file 8: Figure S3b) the resulting RF distance between the two phylogenies is the highest recovered (0.181), though still rather low.

### Supermatrix resampling

**Fig. 3:**
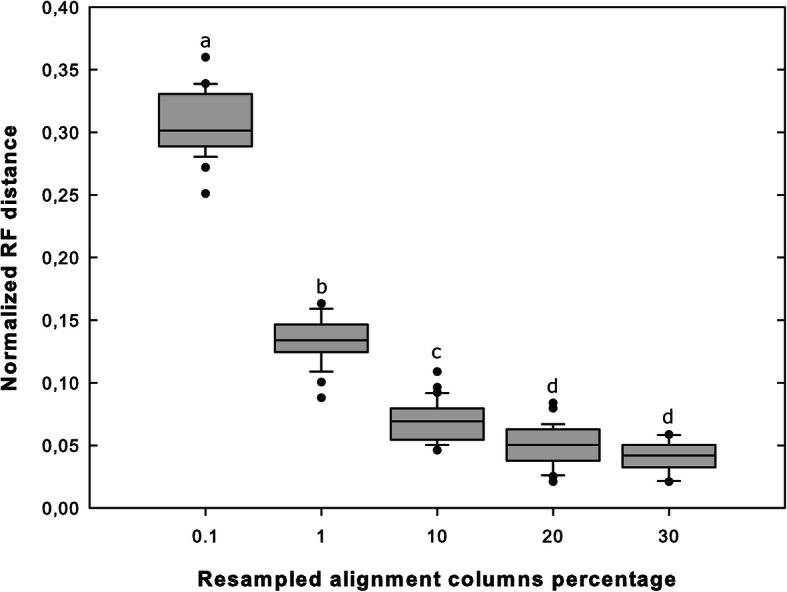
Distribution of the 30 normalized RF distances from the reference topology for each of the five resampling efforts. Boxes are delimited by the distance between the 25th and 75th percentile; lines inside the boxes show the median value of the distribution; whiskers refer to 10th and 90th percentiles; outliers are marked with black dots. Letters (**a**, **b**, **c**, **d**) are used to label statistical significance; boxes with different letters are significantly different (*p* < 0.01) while the same letter indicates no statistical difference

Phylogenetic analyses based on the randomly resampled, increasingly bigger alignment from “1Kb Gblocks” produced topologies which progressively approached the reference (Fig. 3). The results show both an increase of precision and accuracy when the sampling effort is increased. The increase in precision is highlighted by the RF distance among the same dimension resampled matrix topologies, becoming smaller as the amount of resampled columns is increased (Additional file 4: Table S4). The increase of accuracy is shown by the RF distance from the reference topology becoming progressively smaller (concatenation of “1Kb Gblocks” dataset; Fig. 3). The standard deviation (SD) also decreases from 0.026 (0.1% resampling effort) to 0.011 (30% resampling effort), highlighting a smaller distribution variance when the sampling effort is increased. ANOVA and post hoc tests show significant differences among increasing resampling effort up to 20% (ANOVA *p* < 0.01; post hoc tests p < 0.01); conversely, increasing the resampling effort from 20 to 30% did not produce a significant shift of the distances from the reference topology.

### Ancestral character state reconstruction

Maximum parsimony analysis highlighted the rock-inhabiting lifestyle as the most parsimonious state for the basal node of the phylogeny (Table 2). The Maximum Likelihood approach estimated, instead, the saprotrophic lifestyle as the state with the best likelihood (− 185.67), while the rock-inhabiting lifestyle was assigned the second best likelihood score (− 186.41); all the other lifestyles are estimated with a remarkably lower likelihood value (Table 2).

**Table 2:** Ancestral character state reconstruction Log Likelihood values for the basal node of the “>1Kb Gblocks” dataset phylogeny. The states estimated to be the best according to the Likelihood threshold are marked with an asterisk. LIC: lichen; RIF: rock-inhabiting fungus; PP: plant pathogen; SAP: saprotroph; EPI: epiphyte; AP: animal pathogen; FP: fungi pathogen; ECT: ectomycorrhiza; END: endophyte; EXT: extremophile

Lifestyle	Log Likelihood
LIC	− 190.27
RIF	−186.41*
PP	− 189.95
SAP	−185.67*
EPI	−190.50
AP	−190.51
FP	−190.53
ECT	−190.53
END	−190.53
EXT	−190.51

## TAXONOMY

Not applicable.

## DISCUSSION AND CONCLUSIONS

The phylogenomic analyses presented here provide robust insight into evolutionary relationships within Dothideomycetes, with focus on the RIF genera *Lichenothelia* and *Saxomyces*. Exploring different combinations of markers, alignment filtering and phylogenetic reconstruction methods helped to find support among the overarching phylogenetic relationships. Our data suggest that the rock-inhabiting lifestyle is highly likely to be the ancestral state within this diverse class of fungi.

### Supermatrix resampling and phylogenomic inference

Phylogenies are increasingly inferred from datasets comprising unprecedented numbers of samples and genetic markers. However, the number of samples and genetic loci, which both drive the accuracy of phylogenetic inferences, are often far from being comprehensive. Further, which of the two contributes more than the other in the phylogenetic accuracy remains debated, although empirical evidence tends to support the importance of extensive species sampling (Delsuc et al. 2005 and references therein).

Our taxon sampling of Dothideomycetes was determined by the availability of genomes, and we focused our attention on how the differences in the amount of information used for phylogenomic reconstructions may affect tree topology and nodal support. While the results of the resampling experiment should not be generalized, they provide insight into the robustness of phylogenomic inference. Even if samples with a high amount of missing data (up to 90%) were included, we showed that a subset of the whole supermatrix, corresponding to about 20% of our dataset, provides a phylogenetic accuracy which did not improve significantly by further increasing the amount of data (Fig. 3). Moreover, the range of the RF distance of the 20% resampling effort distribution is even smaller than that registered when only the phylogenetic reconstruction method is changed on the complete supermatrix.

Strict alignment filtering criteria have been shown to worsen single-locus inference (Tan et al. 2015). However, in our analyses, strict filtering criteria did not significantly affect the resulting topologies when the length of each locus in the alignment was longer than 1 kb. We adopted this threshold arbitrarily, without testing the effect of a progressively reduced length of each single marker on the resulting phylogenies. Though the effect of the alignment filtering was tested using a strict filtering strategy (Gblocks) and a relaxed strategy (GUIDANCE), we noticed that RF distances between phylogenies, whose pipelines only differ by the filtering step, are among the smallest registered (0.063 and 0.025; Additional file 2: Table S2). This highlights the stability of the phylogenetic signal in our genome-scale data in spite of differences in filtering strategies. In a similar way, even if diverse phylogenetic reconstruction methods were implemented, they produced consistent phylogenies with low RF values, supporting the utility of concatenation-based phylogenomic inference methods, in particular relating to taxon sampling and lineage sorting conditions of our dataset.

Concatenation approaches have been shown to produce highly supported but wrong topologies when sufficiently short branch lengths are generated in relation to effective population size (Kubatko and Degnan 2007). In the present study, coalescent-based approaches were not applied on the whole set of markers because single-locus inferences were heavily affected either by difficult-to-align regions or by strict filtering criteria. The latter would highly reduce the marker length and consequently their phylogenetic signal, leading to single-locus phylogenies dominated by stochastic error (Jeffroy et al. 2006). Here, the coalescent-based method ASTRAL III was applied using 664 individual BUSCO gene topologies (Fig. 2b), resulting in relationships that were largely consistent with the concatenated supermatrix approaches (Fig. 2a). Future, coalescent-based inferences in Dothideomycetes would benefit from a more extensive taxon sampling – a condition not met for many of the species represented by genomic data in this study. Large-scale sequencing projects, such as the “One thousand fungal genomes” (http://1000.fungalgenomes.org) and other laboratories which investigate hidden fungal biodiversity, are filling this gap, sequencing fungal genomes from the least known families. This effort, from a phylogenetic point of view, will make the taxon sampling more comprehensive and, therefore, the phylogenetic inference in Dothideomycetes more accurate.

As we did not find significant topological discrepancies among methods with varying degrees of sensitivity to the noise of phylogenetic signal caused by sequence substitution saturation and/or compositional bias, we refrained from testing targeted filtering approaches (e.g. the exclusion of variable third-codon positions from the alignment) or the use of amino acid alignments instead of nucleotide sequences. The latter would exploit the redundancy of the genetic code to better “preserve” phylogenetic signal (Jeffroy et al. 2006). However, these possible sources of noise should be considered when the most ancient phylogenetic relationships of the tree of life are investigated.

A dataset without any missing data was also tested. In this case, the phylogenetic inferences were reconstructed only based on 63 markers common to all the samples and when certain samples containing high amount of missing data were excluded (e.g. *Rachicladosporium* due to massive gene duplication). We anticipated that this drastic reduction of genetic markers would likely have a greater effect on the resulting phylogeny than the missing data. However, we recovered largely the same topology with the dataset of 664 concatenated genes. Only minor differences concerning the placement of single samples were noted, but these did not involve the most basal nodes. In accordance with other studies which tested real and simulated data (Driskell et al. 2004; Philippe et al. 2004), the phylogenomic dataset assembled here is not negatively affected by missing data, as the incomplete sequences are still represented by enough informative characters.

### Comparison to published Dothideomycetes phylogenies and the phylogenetic placement of RIF

Phylogenomic data have contributed to a shift in our view of evolution, reshuffling many deep evolutionary relationships along the tree of life (Rokas et al. 2003; Fitzpatrick et al. 2006; Wang et al. 2009). In addition, the increasing availability of fungal genomes allows us to investigate evolutionary histories at a finer taxonomic scale. A consistent comparison of tree topologies among previously published phylogenies and the genome-based phylogeny inferred in this study is still not straightforward, because many orders reported in other studies are missing in our phylogenomic inference. Nevertheless, it was possible to detect the main clades which were identified previously and whose phylogenetic position were confirmed by our inference. In general, Dothideomycetes phylogenies published to date identified well-defined lineages at both order and family levels; however, some relationships have remained unsettled. Genome-based inference, as used here, helped to clarify these relationships but cannot be considered the final step to explain all the evolutionary relationships, as limitations concerning the phylogenetic signal of the data and limits of the reconstruction methods can still affect the results. Moreover, sequence availability is still the most relevant bottleneck in phylogenomics, though the scenario is changing quickly.

The subclasses Dothideomycetidae, which includes the orders Capnodiales, Myriangiales and Dothideales, and Pleosporomycetidae, which includes the orders Pleosporales, Mytilinidiales and Hysteriales, have been recovered as highly supported lineages in previous traditional multilocus approaches (Ruibal et al. 2009; Schoch et al. 2009; Muggia et al. 2015; Liu et al. 2017; Ametrano et al. 2019) as well as in the present analyses. Hysteriales and Pleosporales have been recurrently identified as sister clades within Pleosporomycetidae, with the lineage Mytilinidiales basal to them; only Hyde et al. (2013) reported Hysteriales as sister to Mytilinidiales. The relationships of taxa within the subclass Dothideomycetidae is congruent among all the other previous phylogenies and in the present one, confirming Myriangiales and Dothideales as sister groups and Capnodiales as basal to them. The placement of Botryosphaeriales agrees with previous phylogenies (e.g. Ruibal et al. 2009), being basal to the orders belonging to the subclass Pleosporomycetidae. In contrast, the phylogenetic position of Venturiales differs substantially in the present study from other recent multilocus phylogenies produced by Hyde et al. (2013) and Liu et al. (2017). These inferences, indeed, place Venturiales as related to the subclass Dothideomycetidae (e.g. orders Dothideales, Myriangiales and Capnodiales), while our analyses recovered it at the base of the subclass Pleosporomycetidae and order Botryosphaeriales, whereas its relationship with Microthyriales is maintained.

*Lichenothelia* and *Saxomyces* are confirmed to be distinct, independent lineages, as recently reported by Ametrano et al. (2019). However, their placement within Dothideomycetes differs when the inference is based on genomic data. Here *Lichenothelia* samples were recovered as basal to the Dothideomycetes, while *Saxomyces* is early diverging in Dothideomycetidae, diverging from the rest of the clade after *Trypethelium* and *Cryomyces*. The placement of the other two genera of extremotolerant black fungi *Rachicladosporium* and *Hortaea* is congruently found here within Capnodiales, as in previous multilocus phylogenies (Crous et al. 2009). The placement of *Lichenothelia* and *Saxomyces* is particularly interesting because it highlights once more the connection between the lichenized and the not-lichenized lifestyles intrinsic within the two genera (Hawksworth 1981; Muggia et al. 2013, 2015; Ametrano et al. 2017, 2019). It also further strengthens the hypothesis that the earliest diverging taxa of the subclass Dothideomycetidae were rock inhabitants that might have been able to form a lichen-like association with algae. This is in accordance with previous studies where the rock-inhabiting lifestyle was suggested as ancestral for Dothideomycetes and Chaetothyriomycetes (Gueidan et al. 2008). This hypothesis is also supported for Dothideomycetes by our analyses, as both Maximum Parsimony and Maximum Likelihood ancestral character state reconstructions estimate the highest and the second highest score for the rock-inhabiting lifestyle, respectively. The results highlight this lifestyle as ancestral, though taxon sampling within the phylogeny is heavily biased by the lifestyles of those taxa which have been preferentially sequenced due to their economical and biotechnological potential, such as saprotroph and plant pathogens. In the superclass Dothideomyceta, rock inhabitants are also known from Capnodiales (Egidi et al. 2014), of which *Rachicladosporium* is a representative in this study, and Lichenostigmatales (Ertz et al. 2014).

Future studies, investigating character evolution on a more comprehensive genome dataset, representing a more equilibrate proportion of lifestyles within the class, will elucidate the evolution of the different lifestyles in this dynamic fungal class.

## Supplementary information

**Additional file 1: Table S1.** Assemblies retrieved from the NCBI and JGI genome portals and included in the phylogenomic analyses.

**Additional file 2: Table S2.** RF distances and normalized RF distance among the main phylogenies generated from the datasets containing the complete set of samples. Three distances are reported: a regular RF distance and two flavours of weighted RF distance as reported in the RAxML 8.2 manual. Numbers in the first two columns refer to the following starting datasets and reconstruction methods: (0) “> 1 kb Gblocks” IQTree run 1; (1) “> 1 kb Gblocks” IQTree run 2; (2) “> 1 kb Gblocks” IQTree run 3; (3) “> 1 kb Gblocks” ASTRAL; (4) “Complete Gblocks” IQTree; (5) “>1Kb GUIDANCE” IQTree; (6) “>1Kb GUIDANCE” ASTRAL.

**Additional file 3: Table S3.** RF distances and normalized RF distances from the reference topology of the 30 resampled matrices for each resampling effort value (0.1–30%).

**Additional file 4: Table S4.** Average values and standard deviation (SD) of RF distances and normalized RF distances among the 30 phylogenies generated from the randomly resampled matrix with the same resampling effort.

**Additional file 5: Table S5.** Taxa and associated lifestyle used in ancestral character state reconstruction (lichen: 0; rock-inhabiting: 1; plant pathogen: 2; saprotroph: 3; epiphyte: 4; animal pathogen: 5; fungi pathogen: 6; ectomycorrhiza: 7; endophyte: 8; extremophile: 9).

**Additional file 6: Figure S1.** Assembly completeness on the base of 3156 Pezizomycotina orthologs evaluated by BUSCO and expressed as the percentage of complete (green), duplicated (pink) and fragmented or missing (grey) genes. Distribution outliers are highlighted with “*”.

**Additional file 7: Figure S2.** Phylogeny generated from 2998 concatenated genes of the “Complete Gblocks” dataset with IQTree.

**Additional file 8: Figure S3.** Phylogenies generated from 63 genes of the “No missing” dataset; (a) ML tree generated with IQTree on concatenated genes; (b) ASTRAL III species tree.

**Additional file 9: Figure S4.** Phylogeny generated from 1260 concatenated genes of the “>1Kb GUIDANCE” dataset with IQTree.

**Additional file 10: Figure S5.** Phylogeny generated from 1260 concatenated genes of the “>1Kb GUIDANCE” dataset with ASTRAL.

## Data Availability

Alignments used in this study are available at https://zenodo.org/record/3430636#.XYDF0fexVY8. Resampling as well as alignment filtering by length or gap percentage were performed using Python3 scripts (available in GitHub: https://github.com/clof84/Alignment_filtering_and_resampling). Genome assemblies used in this study are available in GenBank and/or JGI Genome portal.

## Abbreviations

ASTRAL: Accurate Species TRee Algorithm
BUSCO: Benchmarking Universal Single Copy Orthologs
CCFEE: Culture Collection of Fungi from Extreme Environments
GUIDANCE: GUIDe tree-based AligNment ConfidencE
JGI: Joint Genome Institute
MAFFT: Multiple Alignment using Fast Fourier Transform
NCBI: National Center for Biotechnology Information
PE: Paired-end
RAxML: Randomized Accelerated Maximum Likelihood
RF: Robinson-Foulds
RIF: Rock inhabiting fungi
