# Supplementary material for: Genome-scale data resolve ancestral rock-inhabiting lifestyle in Dothideomycetes (Ascomycota)
Source: IMA Fungus. 2019 Oct 30;10:19. doi: 10.1186/s43008-019-0018-2 (PMC7325674; doi:10.1186/s43008-019-0018-2)
Supplement: Supplementary file 1 — Additional file 1: Table S1. Assemblies retrieved from the NCBI and JGI genome portals and included in the phylogenomic analyses. [file 43008_2019_18_MOESM1_ESM.docx]

**Table S1.** Assemblies retrieved from NCBI and JGI genome portals and included in the phylogenomic analyses.

| **Species** | **Strain** | **NCBI Accession number, JGI project number** | **Source** | **Submitters/Reference** |
| --- | --- | --- | --- | --- |
| *Aspergillus fischeri* | NRRL 181 | GCA_000149645.2 | NCBI | Fedorova et al. 2008 |
| *Aspergillus fumigatus* | A1163 | GCA_000150145.1 | NCBI | Joardar et al. 2012 |
| *Aspergillus nidulans* | FGSC A4 | GCA_000149205.2 | NCBI | Galagan et al. 2005 |
| *Coccidioides immitis* | RS | GCA_000149335.2 | NCBI | Sharpton et al. 2009 |
| *Histoplasma capsulatum* | var. capsulatum Tmu | GCA_000313325.1 | NCBI | Taipei Medical University, Urology |
| *Paracoccidioides lutzii* | Pb01 | GCA_000150705.2 | NCBI | Desjardins et al. 2011 |
| *Aaosphaeria arxii* | CBS 175.79 | 1019657 | JGI | Joseph Spatafora, Pedro Crous, Janneke Bloem |
| [Acidomyces richmondensis](https://www.ncbi.nlm.nih.gov/Taxonomy/Browser/wwwtax.cgi?mode=Info&id=766039&lvl=3&lin=f&keep=1&srchmode=1&unlock) | BFW | GCA_001592465.1 | NCBI | Mosier et al. 2016 |
| [Acidomyces richmondensis](https://www.ncbi.nlm.nih.gov/Taxonomy/Browser/wwwtax.cgi?mode=Info&id=245562&lvl=3&lin=f&keep=1&srchmode=1&unlock) | meta | GCA_001572075.1 | NCBI | Mosier et al. 2016 |
| *Aliquandostipite khaoyaiensis* | CBS 118232 | 1019641 | JGI | Joseph Spatafora, Jon Karl Magnuson, David E Culley |
| *Alternaria alternata* | 133aPRJ | 1103683 | JGI | Francis Michel Martin, Stéphane Hacquard |
| [Alternaria alternata](https://www.ncbi.nlm.nih.gov/Taxonomy/Browser/wwwtax.cgi?mode=Info&id=5599&lvl=3&lin=f&keep=1&srchmode=1&unlock) | ATCC 34957 | GCA_001443195.1 | NCBI | Nguyen et al. 2016 |
| [Alternaria alternata](https://www.ncbi.nlm.nih.gov/Taxonomy/Browser/wwwtax.cgi?mode=Info&id=5599&lvl=3&lin=f&keep=1&srchmode=1&unlock) | B2a | GCA_001696825.1 | NCBI | Wubetu Bihon, Agricultural Research Organization |
| [Alternaria alternata](https://www.ncbi.nlm.nih.gov/Taxonomy/Browser/wwwtax.cgi?mode=Info&id=5599&lvl=3&lin=f&keep=1&srchmode=1&unlock) | SRC1lrK2f | GCA_001642055.1, 1029430 | NCBI | Zeiner et al. 2016 |
| [Alternaria alternata](https://www.ncbi.nlm.nih.gov/Taxonomy/Browser/wwwtax.cgi?mode=Info&id=5599&lvl=3&lin=f&keep=1&srchmode=1&unlock) | Z7 | GCA_001572055.1 | NCBI | Wang et al. 2016 |
| [Alternaria arborescens](https://www.ncbi.nlm.nih.gov/Taxonomy/Browser/wwwtax.cgi?mode=Info&id=1122867&lvl=3&lin=f&keep=1&srchmode=1&unlock) | EGS 39-128 | GCA_000256225.1 | NCBI | Hu et al. 2012 |
| [Alternaria brassicicola](https://www.ncbi.nlm.nih.gov/Taxonomy/Browser/wwwtax.cgi?mode=Info&id=622360&lvl=3&lin=f&keep=1&srchmode=1&unlock) | ATCC 96836 | GCA_000174375.1 | NCBI | Washington University Genome Center (WUGC), Virginia Bioinformatics Institute |
| [Alternaria consortialis](https://www.ncbi.nlm.nih.gov/Taxonomy/Browser/wwwtax.cgi?mode=Info&id=230004&lvl=3&lin=f&keep=1&srchmode=1&unlock) | JCM 1940 | GCA_001950455.1 | NCBI | RIKEN Center for Life Science Technologies, Division of Genomic Technologies |
| *Amniculicola lignicola* | CBS 123094 | 1011329 | JGI | Joseph Spatafora, Pedro Crous, Janneke Bloem |
| *Ampelomyces quisqualis* | HMLAC05119 | 1051023 | JGI | Joseph Spatafora, Chen Liang |
| *Aplosporella prunicola* | CBS 121.167 | 1006427 | JGI | Joseph Spatafora, Pedro Crous, Charles Cannon |
| [Ascochyta rabiei](https://www.ncbi.nlm.nih.gov/Taxonomy/Browser/wwwtax.cgi?mode=Info&id=5454&lvl=3&lin=f&keep=1&srchmode=1&unlock) | ArDII | GCA_001630375.1 | NCBI | Verma et al. 2016 |
| *Aulographum hederae* | 1006065 | 1006065 | JGI | Joseph Spatafora, Pedro Crous, Lute-Harm Zwiers |
| [Aureobasidium melanogenum](https://www.ncbi.nlm.nih.gov/Taxonomy/Browser/wwwtax.cgi?mode=Info&id=46634&lvl=3&lin=f&keep=1&srchmode=1&unlock) | HN6.2 | GCA_002156615.1 | NCBI | Ocean University of China, Yi Lu |
| [Aureobasidium pullulans](https://www.ncbi.nlm.nih.gov/Taxonomy/Browser/wwwtax.cgi?mode=Info&id=1213350&lvl=3&lin=f&keep=1&srchmode=1&unlock) | AY4 | GCA_000294735.1 | NCBI | Chen et al. 2012 |
| [Aureobasidium pullulans](https://www.ncbi.nlm.nih.gov/Taxonomy/Browser/wwwtax.cgi?mode=Info&id=5580&lvl=3&lin=f&keep=1&srchmode=1&unlock) | IMV 00882 | GCA_001931805.1 | NCBI | Jet Propulsion Laboratory, California Institute of Technology, Kasthuri Venkateswaran |
| [Aureobasidium pullulans](https://www.ncbi.nlm.nih.gov/Taxonomy/Browser/wwwtax.cgi?mode=Info&id=5580&lvl=3&lin=f&keep=1&srchmode=1&unlock) | SAMN04565625 | GCA_001678115.1 | NCBI | Cruz et al. 2016 |
| *Aureobasidium pullulans* var. *melanogenum* | CBS 110374 | GCA_000721775.1, 403630 | JGI | Gosticar et al. 2014 |
| *Aureobasidium pullulans* var. *namibiae* | CBS 147.97 | GCA_000721765.1, 403632 | JGI | Gosticar et al. 2014 |
| *Aureobasidium pullulans* var. *pullulans* | EXF-150 | GCA_000721785.1, 403628 | JGI | Gosticar et al. 2014 |
| *Aureobasidium pullulans* var. *subglaciale* | EXF-2481 | GCA_000721755.1, 403631 | JGI | Gosticar et al. 2014 |
| [*Aureobasidium* sp.](https://www.ncbi.nlm.nih.gov/Taxonomy/Browser/wwwtax.cgi?mode=Info&id=1928478&lvl=3&lin=f&keep=1&srchmode=1&unlock) | FSWF8-4 | GCA_001914275.1 | NCBI | Uppsala University, Sarahi Garcia |
| [Baudoinia panamericana](https://www.ncbi.nlm.nih.gov/Taxonomy/Browser/wwwtax.cgi?mode=Info&id=717646&lvl=3&lin=f&keep=1&srchmode=1&unlock) | UAMH 10762 | GCA_000338955.1 | NCBI | Ohm et al. 2012 |
| [Beverwykella pulmonaria](https://www.ncbi.nlm.nih.gov/Taxonomy/Browser/wwwtax.cgi?mode=Info&id=409207&lvl=3&lin=f&keep=1&srchmode=1&unlock) | JCM 9230 | GCA_001599595.1 | NCBI | RIKEN Center for Life Science Technologies, Division of Genomic Technologies |
| *Bimuria novae-zelandiae* | CBS 107.79 | 1019717 | JGI | Joseph Spatafora, Pedro Crous, Janneke Bloem |
| [Bipolaris maydis](https://www.ncbi.nlm.nih.gov/Taxonomy/Browser/wwwtax.cgi?mode=Info&id=665024&lvl=3&lin=f&keep=1&srchmode=1&unlock) | ATCC 48331 | GCA_000354255.1 | NCBI | Ohm et al. 2012 |
| [Bipolaris maydis](https://www.ncbi.nlm.nih.gov/Taxonomy/Browser/wwwtax.cgi?mode=Info&id=701091&lvl=3&lin=f&keep=1&srchmode=1&unlock) | C5 | GCA_000338975.1 | NCBI | Ohm et al. 2012 |
| [Bipolaris oryzae](https://www.ncbi.nlm.nih.gov/Taxonomy/Browser/wwwtax.cgi?mode=Info&id=930090&lvl=3&lin=f&keep=1&srchmode=1&unlock) | ATCC 44560 | GCA_000523455.1 | NCBI | Condon et al 2013 |
| [Bipolaris oryzae](https://www.ncbi.nlm.nih.gov/Taxonomy/Browser/wwwtax.cgi?mode=Info&id=101162&lvl=3&lin=f&keep=1&srchmode=1&unlock) | TG12bL2 | GCA_001675385.1 | NCBI | University of Minnesota, Juan Gutierrez-Gonzale |
| [Bipolaris sorokiniana](https://www.ncbi.nlm.nih.gov/Taxonomy/Browser/wwwtax.cgi?mode=Info&id=665912&lvl=3&lin=f&keep=1&srchmode=1&unlock) | ND90Pr | GCA_000338995.1 | NCBI | Ohm et al. 2012 |
| [Bipolaris victoriae](https://www.ncbi.nlm.nih.gov/Taxonomy/Browser/wwwtax.cgi?mode=Info&id=930091&lvl=3&lin=f&keep=1&srchmode=1&unlock) | FI3 | GCA_000527765.1 | NCBI | Condon et al 2013 |
| [Bipolaris zeicola](https://www.ncbi.nlm.nih.gov/Taxonomy/Browser/wwwtax.cgi?mode=Info&id=930089&lvl=3&lin=f&keep=1&srchmode=1&unlock) | 26-R-13 | GCA_000523435.1 | NCBI | Condon et al 2013 |
| *Botryosphaeria dothidea* Botdo1 1 | Botdo1 1 | - | JGI | Marsberg et al. 2017 |
| [Botryosphaeria dothidea](https://www.ncbi.nlm.nih.gov/Taxonomy/Browser/wwwtax.cgi?mode=Info&id=55169&lvl=3&lin=f&keep=1&srchmode=1&unlock) | LW030101 | GCA_001717445.1 | NCBI | Qingdao Agricultural University, Sen Lian |
| *Byssothecium circinans* | CBS 675.92 | 1019709 | JGI | Joseph Spatafora, Pedro Crous, Manfred JK Binder |
| [Cenococcum geophilum](https://www.ncbi.nlm.nih.gov/Taxonomy/Browser/wwwtax.cgi?mode=Info&id=794803&lvl=3&lin=f&keep=1&srchmode=1&unlock) | 1.58 | GCA_001692895.1 | NCBI | Peter et al. 2016 |
| [Cercospora canescens](https://www.ncbi.nlm.nih.gov/Taxonomy/Browser/wwwtax.cgi?mode=Info&id=1268270&lvl=3&lin=f&keep=1&srchmode=1&unlock) | BHU | GCA_000347735.1 | NCBI | Institute of Agriculture Sciences, Department of Mycology and Plant Pathology |
| *Cercospora* cf. *sigesbeckiae* | PP 2012 071 | GCA_002217505.1 | NCBI | Louisiana State University, Sebastian Albu |
| [Cercospora sojina](https://www.ncbi.nlm.nih.gov/Taxonomy/Browser/wwwtax.cgi?mode=Info&id=438356&lvl=3&lin=f&keep=1&srchmode=1&unlock) | FLS21 | GCA_002150695.1 | NCBI | Shrestha et al. 2017 |
| [Cercospora sojina](https://www.ncbi.nlm.nih.gov/Taxonomy/Browser/wwwtax.cgi?mode=Info&id=1144355&lvl=3&lin=f&keep=1&srchmode=1&unlock) | S9 | GCA_002084285.1 | NCBI | Zeng et al. 2017 |
| *Cercospora zeae-maydis* | - | 401984 | JGI | Stephen B. Goodwin |
| *Cladosporium fulvum* | CBS 131901 | GCA_000301015.1 | NCBI | Ohm et al. 2012 |
| [Cladosporium sphaerospermum](https://www.ncbi.nlm.nih.gov/Taxonomy/Browser/wwwtax.cgi?mode=Info&id=92950&lvl=3&lin=f&keep=1&srchmode=1&unlock) | IMV 00045 | GCA_001931905.2 | NCBI | Jet Propulsion Laboratory, California Institute of Technology, Kasthuri Venkateswaran |
| [Cladosporium sphaerospermum](https://www.ncbi.nlm.nih.gov/Taxonomy/Browser/wwwtax.cgi?mode=Info&id=1151026&lvl=3&lin=f&keep=1&srchmode=1&unlock) | UM 843 | GCA_000261425.2 | NCBI | Ng et al. 2012 |
| *Clathrospora elynae* | CBS 161.51 | 1019661 | JGI | Joseph Spatafora, Pedro Crous, Manfred JK Binder |
| *Clohesyomyces aquaticus* | CBS 115471 | GCA_002105025.1, 1054410 | NCBI | Mondo et al. 2017 |
| *Cochliobolus heterostrophus* | C5 1 | 52344 | JGI | Ohm et al. 2012 |
| *Cochliobolus lunatus* | m118 | 403758 | JGI | Ohm et al. 2012 |
| [Coniosporium apollinis](https://www.ncbi.nlm.nih.gov/Taxonomy/Browser/wwwtax.cgi?mode=Info&id=1168221&lvl=3&lin=f&keep=1&srchmode=1&unlock) | CBS 100218 | GCA_000281105.1 | NCBI | Teixeira et al. 2017 |
| *Corynespora cassiicola* | CCP | 1019537 | JGI | Lopez et al. 2018 |
| [Corynespora cassiicola](https://www.ncbi.nlm.nih.gov/Taxonomy/Browser/wwwtax.cgi?mode=Info&id=59586&lvl=3&lin=f&keep=1&srchmode=1&unlock) | SAMEA103891068 | GCA_900169545.1 | NCBI | University of Bristol |
| [Corynespora cassiicola](https://www.ncbi.nlm.nih.gov/Taxonomy/Browser/wwwtax.cgi?mode=Info&id=1451142&lvl=3&lin=f&keep=1&srchmode=1&unlock) | UM 591 | GCA_000603925.1 | NCBI | University of Malaya |
| *Cryomyces antarcticus* | CCFEE 534 | GCA_000504465.1 | NCBI | Sterflinger et al. 2014 |
| *Cucurbitaria berberidis* | CBS 394.84 | 1006069 | JGI | Joseph Spatafora, Pedro Crous, Janneke Bloem |
| [Curvularia lunata](https://www.ncbi.nlm.nih.gov/Taxonomy/Browser/wwwtax.cgi?mode=Info&id=1263492&lvl=3&lin=f&keep=1&srchmode=1&unlock) | CX-3 | GCA_000743335.1 | NCBI | Gao et al. 2014 |
| [Curvularia papendorfii](https://www.ncbi.nlm.nih.gov/Taxonomy/Browser/wwwtax.cgi?mode=Info&id=1537989&lvl=3&lin=f&keep=1&srchmode=1&unlock) | UM 226 | GCA_000817285.1 | NCBI | Kuan et al. 2015 |
| [*Curvularia* sp.](https://www.ncbi.nlm.nih.gov/Taxonomy/Browser/wwwtax.cgi?mode=Info&id=1526222&lvl=3&lin=f&keep=1&srchmode=1&unlock) | IFB-Z10 | GCA_002161795.1 | NCBI | Han et al. 2014 |
| *Decorospora gaudefroyi* | P77 CBS 332.63 | 1032355 | JGI | Joseph Spatafora, Patrik Inderbitzin |
| *Delitschia confertaspora* | ATCC 74209 | 1020481 | JGI | Joseph Spatafora, Gerald Bills |
| *Delphinella strobiligena* | CBS 735.71 | 1019673 | JGI | Joseph Spatafora, Jon Karl Magnuson, David E Culley |
| *Didymella exigua* | CBS 183.55 | 407831 | JGI | Joseph Spatafora |
| *Didymella zeae-maydis* | 3018 | - | JGI | Gillian Turgeon |
| *Didymocrea sadasivanii* | CBS 438.65 | 1054428 | JGI | Joseph Spatafora, Jon Karl Magnuson, David E Culley |
| [Diplodia corticola](https://www.ncbi.nlm.nih.gov/Taxonomy/Browser/wwwtax.cgi?mode=Info&id=236234&lvl=3&lin=f&keep=1&srchmode=1&unlock) | CBS 112549 | GCA_001883845.1 | NCBI | University of Aveiro |
| [Diplodia sapinea](https://www.ncbi.nlm.nih.gov/Taxonomy/Browser/wwwtax.cgi?mode=Info&id=1400760&lvl=3&lin=f&keep=1&srchmode=1&unlock) | CMW 190 | GCA_000671355.1 | NCBI | Bihon et al. 2014 |
| [Diplodia sapinea](https://www.ncbi.nlm.nih.gov/Taxonomy/Browser/wwwtax.cgi?mode=Info&id=66738&lvl=3&lin=f&keep=1&srchmode=1&unlock) | CMW39103 | GCA_000729945.1 | NCBI | van der Nest et al. 2014 |
| [Diplodia scrobiculata](https://www.ncbi.nlm.nih.gov/Taxonomy/Browser/wwwtax.cgi?mode=Info&id=280322&lvl=3&lin=f&keep=1&srchmode=1&unlock) | CMW30223 | GCA_001455585.1 | NCBI | Wingfield et al. 2015 |
| [Diplodia seriata](https://www.ncbi.nlm.nih.gov/Taxonomy/Browser/wwwtax.cgi?mode=Info&id=420778&lvl=3&lin=f&keep=1&srchmode=1&unlock) | DS831 | GCA_001006355.1 | NCBI | Morales-Cruz et al. 2015 |
| [Diplodia seriata](https://www.ncbi.nlm.nih.gov/Taxonomy/Browser/wwwtax.cgi?mode=Info&id=420778&lvl=3&lin=f&keep=1&srchmode=1&unlock) | F98.1 | GCA_001975905.1 | NCBI | INRA, Guillaume Robert |
| *Dissoconium aciculare* | CBS 342.82 | 1011337 | JGI | Joseph Spatafora, Pedro Crous, Manfred JK Binder |
| *Dothidotthia symphoricarpi* | CBS 119.687 | 1011345 | JGI | Joseph Spatafora, Pedro Crous, Janneke Bloem |
| [Dothistroma pini](https://www.ncbi.nlm.nih.gov/Taxonomy/Browser/wwwtax.cgi?mode=Info&id=1367539&lvl=3&lin=f&keep=1&srchmode=1&unlock) | CBS 116.487 | GCA_002116355.1 | NCBI | Canada's Michael Smith Genome Sciences Centre |
| [Dothistroma septosporum](https://www.ncbi.nlm.nih.gov/Taxonomy/Browser/wwwtax.cgi?mode=Info&id=64363&lvl=3&lin=f&keep=1&srchmode=1&unlock) | CMW 10211 | GCA_002236575.1 | NCBI | Massey University |
| [Dothistroma septosporum](https://www.ncbi.nlm.nih.gov/Taxonomy/Browser/wwwtax.cgi?mode=Info&id=64363&lvl=3&lin=f&keep=1&srchmode=1&unlock) | CMW 10798 | GCA_002236655.1 | NCBI | Massey University |
| [Dothistroma septosporum](https://www.ncbi.nlm.nih.gov/Taxonomy/Browser/wwwtax.cgi?mode=Info&id=64363&lvl=3&lin=f&keep=1&srchmode=1&unlock) | CMW 11305 | GCA_002236515.1 | NCBI | Massey University |
| [Dothistroma septosporum](https://www.ncbi.nlm.nih.gov/Taxonomy/Browser/wwwtax.cgi?mode=Info&id=64363&lvl=3&lin=f&keep=1&srchmode=1&unlock) | CMW 13121 | GCA_002236675.1 | NCBI | Massey University |
| [Dothistroma septosporum](https://www.ncbi.nlm.nih.gov/Taxonomy/Browser/wwwtax.cgi?mode=Info&id=64363&lvl=3&lin=f&keep=1&srchmode=1&unlock) | CMW 13123 | GCA_002236485.1 | NCBI | Massey University |
| [Dothistroma septosporum](https://www.ncbi.nlm.nih.gov/Taxonomy/Browser/wwwtax.cgi?mode=Info&id=64363&lvl=3&lin=f&keep=1&srchmode=1&unlock) | CMW 14822 | GCA_002236475.1 | NCBI | Massey University |
| [Dothistroma septosporum](https://www.ncbi.nlm.nih.gov/Taxonomy/Browser/wwwtax.cgi?mode=Info&id=64363&lvl=3&lin=f&keep=1&srchmode=1&unlock) | CMW 14823 | GCA_002236775.1 | NCBI | Massey University |
| [Dothistroma septosporum](https://www.ncbi.nlm.nih.gov/Taxonomy/Browser/wwwtax.cgi?mode=Info&id=64363&lvl=3&lin=f&keep=1&srchmode=1&unlock) | CMW 15843 | GCA_002236615.1 | NCBI | Massey University |
| [Dothistroma septosporum](https://www.ncbi.nlm.nih.gov/Taxonomy/Browser/wwwtax.cgi?mode=Info&id=64363&lvl=3&lin=f&keep=1&srchmode=1&unlock) | CMW 23429 | GCA_002236725.1 | NCBI | Massey University |
| [Dothistroma septosporum](https://www.ncbi.nlm.nih.gov/Taxonomy/Browser/wwwtax.cgi?mode=Info&id=64363&lvl=3&lin=f&keep=1&srchmode=1&unlock) | CMW 37193 | GCA_002236645.1 | NCBI | Massey University |
| [Dothistroma septosporum](https://www.ncbi.nlm.nih.gov/Taxonomy/Browser/wwwtax.cgi?mode=Info&id=64363&lvl=3&lin=f&keep=1&srchmode=1&unlock) | CMW 37194 | GCA_002236545.1 | NCBI | Massey University |
| [Dothistroma septosporum](https://www.ncbi.nlm.nih.gov/Taxonomy/Browser/wwwtax.cgi?mode=Info&id=64363&lvl=3&lin=f&keep=1&srchmode=1&unlock) | CMW 37965 | GCA_002236745.1 | NCBI | Massey University |
| [Dothistroma septosporum](https://www.ncbi.nlm.nih.gov/Taxonomy/Browser/wwwtax.cgi?mode=Info&id=64363&lvl=3&lin=f&keep=1&srchmode=1&unlock) | CMW 38941 | GCA_002236925.1 | NCBI | Massey University |
| [Dothistroma septosporum](https://www.ncbi.nlm.nih.gov/Taxonomy/Browser/wwwtax.cgi?mode=Info&id=64363&lvl=3&lin=f&keep=1&srchmode=1&unlock) | CMW 40004 | GCA_002236585.1 | NCBI | Massey University |
| [Dothistroma septosporum](https://www.ncbi.nlm.nih.gov/Taxonomy/Browser/wwwtax.cgi?mode=Info&id=64363&lvl=3&lin=f&keep=1&srchmode=1&unlock) | CMW 44207 | GCA_002236565.1 | NCBI | Massey University |
| [Dothistroma septosporum](https://www.ncbi.nlm.nih.gov/Taxonomy/Browser/wwwtax.cgi?mode=Info&id=64363&lvl=3&lin=f&keep=1&srchmode=1&unlock) | CMW 44656 | GCA_002236685.1 | NCBI | Massey University |
| [Dothistroma septosporum](https://www.ncbi.nlm.nih.gov/Taxonomy/Browser/wwwtax.cgi?mode=Info&id=64363&lvl=3&lin=f&keep=1&srchmode=1&unlock) | MU NZE8 | GCA_002236465.1 | NCBI | Massey University |
| *Dothistroma septosporum* | NZE10 v1.0 | GCA_000340195.1 | NCBI | Ohm et al. 2012 |
| [Dothistroma septosporum](https://www.ncbi.nlm.nih.gov/Taxonomy/Browser/wwwtax.cgi?mode=Info&id=64363&lvl=3&lin=f&keep=1&srchmode=1&unlock) | NZFS4520 | GCA_002236755.1 | NCBI | Massey University |
| *Elsinoe ampelina* CECT 20119 | CECT 20119 | 1064684 | JGI | Joseph Spatafora, Manuel Alfaro Sánchez |
| [Epicoccum nigrum](https://www.ncbi.nlm.nih.gov/Taxonomy/Browser/wwwtax.cgi?mode=Info&id=105696&lvl=3&lin=f&keep=1&srchmode=1&unlock) | ICMP 19927 | GCA_002116315.1 | NCBI | The University of Auckland |
| [Epicoccum sorghinum](https://www.ncbi.nlm.nih.gov/Taxonomy/Browser/wwwtax.cgi?mode=Info&id=749593&lvl=3&lin=f&keep=1&srchmode=1&unlock) | USPMTOX48 | GCA_001879705.1 | NCBI | University of Sao Paulo |
| *Eremomyces bilateralis* | CBS 781.70 | 1011349 | JGI | Joseph Spatafora, Pedro Crous, Manfred JK Binder |
| [Glonium stellatum](https://www.ncbi.nlm.nih.gov/Taxonomy/Browser/wwwtax.cgi?mode=Info&id=574774&lvl=3&lin=f&keep=1&srchmode=1&unlock) | CBS 207.34 | GCA_001692915.1 | NCBI | Spatafora et al. 2012 |
| [Helminthosporium solani](https://www.ncbi.nlm.nih.gov/Taxonomy/Browser/wwwtax.cgi?mode=Info&id=1400345&lvl=3&lin=f&keep=1&srchmode=1&unlock) | B-AC-16A | GCA_000498615.1 | NCBI | University of Wisconsin-Madison |
| *Hortaea acidophila* | CBS 113389 | 1040524 | JGI | Joseph Spatafora, Jon Karl Magnuson |
| *Hortaea werneckii* | EXF-2000 | GCA_002127715.1 | NCBI | University of California, Riverside |
| *Hortaea werneckii* | EXF-2000M0 scaffolds | GCA_000410955.1 | NCBI | Lenassi et al. 2013 |
| *Hysterium pulicare* | CBS 123377 | GCA_000467715.1 | NCBI | Ohm et al. 2012 |
| *Karstenula rhodostoma* | CBS 690.94 | 1019721 | JGI | Joseph Spatafora, Pedro Crous, Janneke Bloem |
| [Lasiodiplodia theobromae](https://www.ncbi.nlm.nih.gov/Taxonomy/Browser/wwwtax.cgi?mode=Info&id=45133&lvl=3&lin=f&keep=1&srchmode=1&unlock) | CSS-01s | GCA_002111425.1 | NCBI | Beijing Academy of Agriculture and Forestry Sciences |
| [Lecanosticta acicola](https://www.ncbi.nlm.nih.gov/Taxonomy/Browser/wwwtax.cgi?mode=Info&id=1367079&lvl=3&lin=f&keep=1&srchmode=1&unlock) | CBS 871.95 | GCA_000504345.2 | NCBI | Canada's Michael Smith Genome Sciences Centre |
| *Lentithecium fluviatile* | CBS 122.367 | 1006093 | JGI | Joseph Spatafora, Pedro Crous, Janneke Bloem |
| [Lepidopterella palustris](https://www.ncbi.nlm.nih.gov/Taxonomy/Browser/wwwtax.cgi?mode=Info&id=1314670&lvl=3&lin=f&keep=1&srchmode=1&unlock) | CBS 459.81 | GCA_001692735.1 | NCBI | Peter et al.2016 |
| [Leptosphaeria maculans](https://www.ncbi.nlm.nih.gov/Taxonomy/Browser/wwwtax.cgi?mode=Info&id=985895&lvl=3&lin=f&keep=1&srchmode=1&unlock) | JN3 | GCA_000230375.1 | NCBI | Rouxel et al. 2011 |
| *Leptosphaeria maculans* | NZT4 | GCA_900465115.1 | NCBI | Genomic Standards Consortium |
| [Leptoxyphium fumago](https://www.ncbi.nlm.nih.gov/Taxonomy/Browser/wwwtax.cgi?mode=Info&id=5474&lvl=3&lin=f&keep=1&srchmode=1&unlock) | SC3815 | GCA_001660795.1 | NCBI | International Institute Zittau - TU Dresden, Harald Kellner |
| *Lindgomyces ingoldianus* | ATCC 200398 | 1042879 | JGI | Joseph Spatafora, Jon Karl Magnuson, David E Culley |
| *Lineolata rhizophorae* | ATCC16933 | 1051209 | JGI | Joseph Spatafora, Jon Karl Magnuson, David E Culley |
| *Lizonia empirigonia* | CBS 542.76 | 1019757 | JGI | Joseph Spatafora, Pedro Crous, Manfred JK Binder |
| *Lophiostoma macrostomum* | CBS 122.681 | 1011357 | JGI | Joseph Spatafora, Pedro Crous, Manfred JK Binder |
| *Lophiotrema nucula* | CBS 627.86 | 1019701 | JGI | Joseph Spatafora, Jon Karl Magnuson, David E Culley |
| *Lophium mytilinum* | CBS 269.34 | 1019725 | JGI | Joseph Spatafora, Pedro Crous, Janneke Bloem |
| [Macrophomina phaseolina](https://www.ncbi.nlm.nih.gov/Taxonomy/Browser/wwwtax.cgi?mode=Info&id=35725&lvl=3&lin=f&keep=1&srchmode=1&unlock) | MO00014 | GCA_001307885.1 | NCBI | University of Illinois, Gloria Rendon |
| [Macrophomina phaseolina](https://www.ncbi.nlm.nih.gov/Taxonomy/Browser/wwwtax.cgi?mode=Info&id=35725&lvl=3&lin=f&keep=1&srchmode=1&unlock) | MP00003 | GCA_001307925.1 | NCBI | University of Illinois, Gloria Rendon |
| [Macrophomina phaseolina](https://www.ncbi.nlm.nih.gov/Taxonomy/Browser/wwwtax.cgi?mode=Info&id=35725&lvl=3&lin=f&keep=1&srchmode=1&unlock) | MP00065 | GCA_001307945.1 | NCBI | University of Illinois, Gloria Rendon |
| [Macrophomina phaseolina](https://www.ncbi.nlm.nih.gov/Taxonomy/Browser/wwwtax.cgi?mode=Info&id=35725&lvl=3&lin=f&keep=1&srchmode=1&unlock) | MP00325 | GCA_001307935.1 | NCBI | University of Illinois, Gloria Rendon |
| [Macrophomina phaseolina](https://www.ncbi.nlm.nih.gov/Taxonomy/Browser/wwwtax.cgi?mode=Info&id=35725&lvl=3&lin=f&keep=1&srchmode=1&unlock) | MP00327 | GCA_001307955.1 | NCBI | University of Illinois, Gloria Rendon |
| [Macrophomina phaseolina](https://www.ncbi.nlm.nih.gov/Taxonomy/Browser/wwwtax.cgi?mode=Info&id=35725&lvl=3&lin=f&keep=1&srchmode=1&unlock) | MRf1 | GCA_001051165.1 | NCBI | Junagadh Agricultural University, Manoj Parakhia |
| [Macrophomina phaseolina](https://www.ncbi.nlm.nih.gov/Taxonomy/Browser/wwwtax.cgi?mode=Info&id=1126212&lvl=3&lin=f&keep=1&srchmode=1&unlock) | MS6 | GCA_000302655.1 | NCBI | Islam et al. 2012 |
| *Macroventuria anomochaeta* | CBS 525.71 | 1019665 | JGI | Joseph Spatafora, Pedro Crous, Janneke Bloem |
| *Massarina eburnea* | CBS 473.64 | 1011361 | JGI | Joseph Spatafora, Pedro Crous, Manfred JK Binder |
| *Massariosphaeria phaeospora* | CBS 611.86 | 1019705 | JGI | Joseph Spatafora, Jon Karl Magnuson, David E Culley |
| *Melanomma pulvis-pyrius* | 1011365 | 1011365 | JGI | Joseph Spatafora, Pedro Crous, Manfred JK Binder |
| *Microthyrium microscopicum* | CBS 115976 | 1011369 | JGI | Joseph Spatafora, Pedro Crous, Manfred JK Binder |
| [Mycosphaerella arachidis](https://www.ncbi.nlm.nih.gov/Taxonomy/Browser/wwwtax.cgi?mode=Info&id=143450&lvl=3&lin=f&keep=1&srchmode=1&unlock) | CALF-13A | GCA_001297265.1 | NCBI | Orner et al. 2015 |
| *Mycosphaerella eumusae* | CBS 114824 | GCA_001578235.1 | NCBI | Chang et al. 2016 |
| *Mycosphaerella graminicola* | - | 16205 | JGI | Goodwin et al. 2011 |
| [Mycosphaerella laricina](https://www.ncbi.nlm.nih.gov/Taxonomy/Browser/wwwtax.cgi?mode=Info&id=1367536&lvl=3&lin=f&keep=1&srchmode=1&unlock) | CBS 326.52 | GCA_000504385.2 | NCBI | Canada's Michael Smith Genome Sciences Centre |
| *Mycosphaerella* sp. | PB-2012b Mex 2-1-2 | GCA_002116345.1 | NCBI | Canada's Michael Smith Genome Sciences Centre |
| *Mycosphaerella* sp. | Ston1 | GCA_000504405.2 | NCBI | Canada's Michael Smith Genome Sciences Centre |
| *Myriangium duriaei* | CBS 260.36 | 1006105 | JGI | Joseph Spatafora, Pedro Crous, Janneke Bloem |
| *Mytilinidion resinicola* | CBS 304.34 | 1040537 | JGI | Joseph Spatafora, Jon Karl Magnuson, David E Culley |
| [Neofusicoccum parvum](https://www.ncbi.nlm.nih.gov/Taxonomy/Browser/wwwtax.cgi?mode=Info&id=1287680&lvl=3&lin=f&keep=1&srchmode=1&unlock) | UCRNP2 | GCA_000385595.1 | NCBI | Blanco-Ulate et al. 2013 |
| *Nigrograna mackinnonii* | E5202H | GCA_001007845.1 | NCBI | Shaw et al. 2015 |
| [Ochroconis constricta](https://www.ncbi.nlm.nih.gov/Taxonomy/Browser/wwwtax.cgi?mode=Info&id=1442076&lvl=3&lin=f&keep=1&srchmode=1&unlock) | UM 578 | GCA_000611715.1 | NCBI | Chan et al. 2014 |
| *Ophiobolus disseminans* | CBS 113818 | 1019733 | JGI | Joseph Spatafora, Pedro Crous, Manfred JK Binder |
| [Paraphaeosphaeria sporulosa](https://www.ncbi.nlm.nih.gov/Taxonomy/Browser/wwwtax.cgi?mode=Info&id=1460663&lvl=3&lin=f&keep=1&srchmode=1&unlock) | AP3s5-JAC2a | 1029422 | JGI | Zeinier at al. 2016 |
| [*Paraphoma* sp.](https://www.ncbi.nlm.nih.gov/Taxonomy/Browser/wwwtax.cgi?mode=Info&id=1804209&lvl=3&lin=f&keep=1&srchmode=1&unlock) | B47-9 | GCA_001748405.1 | NCBI | National Institute for Agro-Environmental Sciences (NIAES) |
| *Parastagonospora nodorum* | SN15 | GCA_000146915.2 | NCBI | National Institute for Agro-Environmental Sciences (NIAES) |
| *Patellaria atrata* | CBS 101060 | 1006113 | JGI | Joseph Spatafora, Pedro Crous, Janneke Bloem |
| [Peltaster fructicola](https://www.ncbi.nlm.nih.gov/Taxonomy/Browser/wwwtax.cgi?mode=Info&id=286661&lvl=3&lin=f&keep=1&srchmode=1&unlock) | LNHT1506 | GCA_001592805.1 | NCBI | Xu et al. 2016 |
| *Periconia macrospinosa* | DSE2036 | 1025594 | JGI | Knapp et al. 2018 |
| [Phaeocryptopus gaeumannii](https://www.ncbi.nlm.nih.gov/Taxonomy/Browser/wwwtax.cgi?mode=Info&id=1367540&lvl=3&lin=f&keep=1&srchmode=1&unlock) | CBS 267.37 | GCA_002116385.1 | NCBI | Canada's Michael Smith Genome Sciences Centre |
| *Phaeosphaeria nodorum* | Sn79 1.0 | GCA_002216185.1 | NCBI | Canada's Michael Smith Genome Sciences Centre |
| Phaeosphaeriaceae sp. | PMI 808 | 1021562 | JGI | Joseph Spatafora, Gregory Bonito, Hui-Ling Liao |
| [Phoma herbarum](https://www.ncbi.nlm.nih.gov/Taxonomy/Browser/wwwtax.cgi?mode=Info&id=73001&lvl=3&lin=f&keep=1&srchmode=1&unlock) | JCM 15942 | GCA_001599375.1 | NCBI | RIKEN Center for Life Science Technologies, Division of Genomic Technologies |
| *Phoma tracheiphila* | IPT5 | 1021241 | JGI | Joseph Spatafora, David Ezra |
| *Phyllosticta capitalensis* | CBS 128.856 | 1109085 | JGI | Francis Michel Martin, Vladimiro Guarnaccia |
| *Phyllosticta capitalensis* | Gm33 | GCA_001604925.1 | NCBI | RIKEN Center for Life Science Technologies, Division of Genomic Technologies |
| *Phyllosticta citriasiana* | CBS 120486 | 1011301 | JGI | Joseph Spatafora, Pedro Crous, Manfred JK Binder |
| *Phyllosticta citribraziliensis* | CBS 100098 | 1109089 | JGI | Francis Michel Martin, Vladimiro Guarnaccia |
| [Phyllosticta citricarpa](https://www.ncbi.nlm.nih.gov/Taxonomy/Browser/wwwtax.cgi?mode=Info&id=1291520&lvl=3&lin=f&keep=1&srchmode=1&unlock) | CGMCC3.14348 | GCA_000382785.1 | NCBI | Zhejiang university |
| [Phyllosticta citricarpa](https://www.ncbi.nlm.nih.gov/Taxonomy/Browser/wwwtax.cgi?mode=Info&id=55181&lvl=3&lin=f&keep=1&srchmode=1&unlock) | Gc12 | GCA_001604955.1 | NCBI | Citrus Research and Education Center, University of Florida |
| *Phyllosticta citrichinaensis* | CBS 130529 | 1109091 | JGI | Francis Michel Martin, Vladimiro Guarnaccia |
| *Phyllosticta* sp. | CPC 27169 | 1109095 | JGI | Francis Michel Martin, Vladimiro Guarnaccia |
| *Phyllosticta* sp. | CPC 27913 | 1109093 | JGI | Francis Michel Martin, Vladimiro Guarnaccia |
| *Piedraia hortae* | CBS 480.64 | 1011305 | JGI | Joseph Spatafora, Pedro Crous, Janneke Bloem |
| *Pleomassaria siparia* | CBS279.74 | 1011309 | JGI | Joseph Spatafora, Pedro Crous, Manfred JK Binder |
| [*Pleosporales* sp.](https://www.ncbi.nlm.nih.gov/Taxonomy/Browser/wwwtax.cgi?mode=Info&id=1162309&lvl=3&lin=f&keep=1&srchmode=1&unlock) | UM 1110 | GCA_000263175.2 | NCBI | Ng et al. 2012 |
| *Polychaeton citri* | - | 1011313 | JGI | Joseph Spatafora, Pedro Crous, Manfred JK Binder |
| *Polyplosphaeria fusca* | CBS 125425 | 1019777 | JGI | Joseph Spatafora, Pedro Crous, Manfred JK Binder |
| *Preussia* sp. | BSL10 | GCA_001553865.1 | NCBI | University of Nizwa, Abdul Khan |
| *Pseudocercospora fijiensis* | CIRAD86 | GCA_000340215.1 | NCBI | Ohm et al. 2012 |
| [Pseudocercospora musae](https://www.ncbi.nlm.nih.gov/Taxonomy/Browser/wwwtax.cgi?mode=Info&id=113226&lvl=3&lin=f&keep=1&srchmode=1&unlock) | CBS 116634 | GCA_001578225.1 | NCBI | Chang et al. 2016 |
| [Pseudocercospora pini-densiflorae](https://www.ncbi.nlm.nih.gov/Taxonomy/Browser/wwwtax.cgi?mode=Info&id=1367541&lvl=3&lin=f&keep=1&srchmode=1&unlock) | CBS 125139 | GCA_000504365.2 | NCBI | Canada's Michael Smith Genome Sciences Centre |
| *Pseudovirgaria hyperparasitica* | CBS 121739 | 1032442 | JGI | Joseph Spatafora, Pedro Crous, Manfred JK Binder |
| [Pyrenochaeta lycopersici](https://www.ncbi.nlm.nih.gov/Taxonomy/Browser/wwwtax.cgi?mode=Info&id=1331946&lvl=3&lin=f&keep=1&srchmode=1&unlock) | CRA-PAV ER 1211 | GCA_000601435.1 | NCBI | Aragona et al. 2014 |
| [*Pyrenochaeta* sp.](https://www.ncbi.nlm.nih.gov/Taxonomy/Browser/wwwtax.cgi?mode=Info&id=765867&lvl=3&lin=f&keep=1&srchmode=1&unlock) | DS3sAY3a | GCA_001644535.1 | NCBI | Zeiner et al. 2016 |
| [*Pyrenochaeta* sp.](https://www.ncbi.nlm.nih.gov/Taxonomy/Browser/wwwtax.cgi?mode=Info&id=765867&lvl=3&lin=f&keep=1&srchmode=1&unlock) | UM 256 | GCA_000359685.1 | NCBI | Yew et al. 2013 |
| *Pyrenophora seminiperda* | CCB06 | GCA_000465215.1 | NCBI | Soliai et al. 2014 |
| *Pyrenophora teres* | f. teres 0-1 | GCA_000166005.1 | NCBI | Ellwood et al. 2010 |
| *Pyrenophora tritici-repentis* | Pt-1C-BFP | GCA_000149985.1 | NCBI | Broad Institute of MIT and Harvard |
| [Rachicladosporium antarcticum](https://www.ncbi.nlm.nih.gov/Taxonomy/Browser/wwwtax.cgi?mode=Info&id=1507870&lvl=3&lin=f&keep=1&srchmode=1&unlock) | CCFEE 5527 | GCA_002077065.1 | NCBI | Coleine et al. 2017 |
| [*Rachicladosporium* sp.](https://www.ncbi.nlm.nih.gov/Taxonomy/Browser/wwwtax.cgi?mode=Info&id=1974281&lvl=3&lin=f&keep=1&srchmode=1&unlock) | CCFEE 5018 | GCA_002077045.2 | NCBI | Coleine et al. 2017 |
| [Ramularia collo-cygni](https://www.ncbi.nlm.nih.gov/Taxonomy/Browser/wwwtax.cgi?mode=Info&id=112498&lvl=3&lin=f&keep=1&srchmode=1&unlock) | DK05 | GCA_001510955.1 | NCBI | European Bioinformatics Institute (EBI) |
| [Ramularia endophylla](https://www.ncbi.nlm.nih.gov/Taxonomy/Browser/wwwtax.cgi?mode=Info&id=1367538&lvl=3&lin=f&keep=1&srchmode=1&unlock) | CBS 113265 | GCA_002116395.1 | NCBI | Canada's Michael Smith Genome Sciences Centre |
| *Rhizodiscina lignyota* | CBS 133067 | 1019729 | JGI | Joseph Spatafora, Pedro Crous, Janneke Bloem |
| *Rhytidhysteron rufulum* | CBS 306.38 | GCA_000467735.1, 1019729 | NCBI | Ohm et al. 2012 |
| *Saccharata proteae* | CBS 121410 | 1011317 | JGI | Joseph Spatafora, Pedro Crous, Manfred JK Binder |
| *Setomelanomma holmii* | CBS 110217 | 1019737 | JGI | Joseph Spatafora, Pedro Crous, Manfred JK Binder |
| [Setosphaeria turcica](https://www.ncbi.nlm.nih.gov/Taxonomy/Browser/wwwtax.cgi?mode=Info&id=671987&lvl=3&lin=f&keep=1&srchmode=1&unlock) | Et28A | GCA_000359705.1 | NCBI | Ohm et al. 2012 |
| *Setosphaeria turcica* | NY001 | 1036047 | JGI | Gerrit HJ Kema, Gillian Turgeon |
| *Shiraia* sp. | slf14 | GCA_000498155.1 | NCBI | Yang et al. 2014 |
| [Sphaerulina musiva](https://www.ncbi.nlm.nih.gov/Taxonomy/Browser/wwwtax.cgi?mode=Info&id=692275&lvl=3&lin=f&keep=1&srchmode=1&unlock) | SO2202 | GCA_000320565.2 | NCBI | Ohm et al. 2012 |
| [Sphaerulina populicola](https://www.ncbi.nlm.nih.gov/Taxonomy/Browser/wwwtax.cgi?mode=Info&id=1136490&lvl=3&lin=f&keep=1&srchmode=1&unlock) | P02.02b | GCA_000291705.1 | NCBI | University of British Columbia, Forest Sciences |
| *Sporormia fimetaria* | CBS 119.925 | 1011321 | JGI | Joseph Spatafora, Pedro Crous, Manfred JK Binder |
| *Stagonospora* sp. | SRC1lsM3a | GCA_001644525.1 | NCBI | Zeiner et al. 2016 |
| [Stagonosporopsis tanaceti](https://www.ncbi.nlm.nih.gov/Taxonomy/Browser/wwwtax.cgi?mode=Info&id=1200839&lvl=3&lin=f&keep=1&srchmode=1&unlock) | CBS 131484 | GCA_000812845.1 | NCBI | Vaghefi et al. 2015 |
| [Stemphylium lycopersici](https://www.ncbi.nlm.nih.gov/Taxonomy/Browser/wwwtax.cgi?mode=Info&id=183478&lvl=3&lin=f&keep=1&srchmode=1&unlock) | CIDEFI 216 | GCA_001191545.1 | NCBI | Franco et al. 2015 |
| *Teratosphaeria nubilosa* | CBS 116005 | 1019765 | JGI | Joseph Spatafora, Pedro Crous, Manfred JK Binder |
| *Tothia fuscella* | CBS 130266 | 1019713 | JGI | Joseph Spatafora, Pedro Crous, Manfred JK Binder |
| *Trematosphaeria pertusa* | CBS 122368 | 1019781 | JGI | Joseph Spatafora, Pedro Crous, Manfred JK Binder |
| *Trichodelitschia bisporula* | CBS 262.69 | 1019741 | JGI | Joseph Spatafora, Pedro Crous, Manfred JK Binder |
| *Trypethelium eluteriae* | - | 1006394 | JGI | Stephen Goodwin, Daniele Armaleo |
| [Venturia carpophila](https://www.ncbi.nlm.nih.gov/Taxonomy/Browser/wwwtax.cgi?mode=Info&id=86257&lvl=3&lin=f&keep=1&srchmode=1&unlock) | JP3-5 | GCA_001990985.1 | NCBI | USDA-ARS, Chunxian Chen |
| [Venturia effusa](https://www.ncbi.nlm.nih.gov/Taxonomy/Browser/wwwtax.cgi?mode=Info&id=50376&lvl=3&lin=f&keep=1&srchmode=1&unlock) | 3Des10b | GCA_001901625.1 | NCBI | Bock et al. 2016 |
| [Venturia inaequalis](https://www.ncbi.nlm.nih.gov/Taxonomy/Browser/wwwtax.cgi?mode=Info&id=5025&lvl=3&lin=f&keep=1&srchmode=1&unlock) | 1389 | GCA_002148275.1 | NCBI | Shiller et al. 2015 |
| [Venturia inaequalis](https://www.ncbi.nlm.nih.gov/Taxonomy/Browser/wwwtax.cgi?mode=Info&id=5025&lvl=3&lin=f&keep=1&srchmode=1&unlock) | 1639 | GCA_002148295.1 | NCBI | Shiller et al. 2015 |
| [Venturia inaequalis](https://www.ncbi.nlm.nih.gov/Taxonomy/Browser/wwwtax.cgi?mode=Info&id=5025&lvl=3&lin=f&keep=1&srchmode=1&unlock) | EU-B04 | GCA_002148305.1 | NCBI | Shiller et al. 2015 |
| *Venturia inaequalis* | ICMP 13258 | GCA_001857725.1 | NCBI | Shiller et al. 2015 |
| *Venturia pyrina* | ICMP 11032 | GCA_000738655.1 | NCBI | Cooke et al. 2014 |
| [Verruconis gallopava](https://www.ncbi.nlm.nih.gov/Taxonomy/Browser/wwwtax.cgi?mode=Info&id=253628&lvl=3&lin=f&keep=1&srchmode=1&unlock) | CBS 43764 | GCA_000836295.1 | NCBI | Broad Institute, Sinead Chapman |
| *Verruculina enalia* | CBS 304.66 | 1019669 | JGI | Joseph Spatafora, Pedro Crous, Janneke Bloem |
| *Westerdykella ornata* | CBS 379.55 | 1019761 | JGI | Joseph Spatafora, Pedro Crous, Manfred JK Binder |
| *Zasmidium cellare* | ATCC 36951 | 402903 | JGI | Stephen B. Goodwin |
| *Zopfia rhizophila* | - | 1011325 | JGI | Joseph Spatafora, Pedro Crous, Janneke Bloem |
| [Zymoseptoria ardabiliae](https://www.ncbi.nlm.nih.gov/Taxonomy/Browser/wwwtax.cgi?mode=Info&id=1173716&lvl=3&lin=f&keep=1&srchmode=1&unlock) | ST11IR 6.1.1 | GCA_000983525.1 | NCBI | Grandaubert et al. 2015 |
| [Zymoseptoria ardabiliae](https://www.ncbi.nlm.nih.gov/Taxonomy/Browser/wwwtax.cgi?mode=Info&id=985147&lvl=3&lin=f&keep=1&srchmode=1&unlock) | STIR04 1.1.1 | GCA_000223745.2 | NCBI | Stukenbrock et al. 2011 |
| [Zymoseptoria ardabiliae](https://www.ncbi.nlm.nih.gov/Taxonomy/Browser/wwwtax.cgi?mode=Info&id=985145&lvl=3&lin=f&keep=1&srchmode=1&unlock) | STIR04 1.1.2 | GCA_000223765.2 | NCBI | Stukenbrock et al. 2011 |
| [Zymoseptoria ardabiliae](https://www.ncbi.nlm.nih.gov/Taxonomy/Browser/wwwtax.cgi?mode=Info&id=985144&lvl=3&lin=f&keep=1&srchmode=1&unlock) | STIR04 3.13.1 | GCA_000223785.2 | NCBI | Stukenbrock et al. 2011 |
| [Zymoseptoria ardabiliae](https://www.ncbi.nlm.nih.gov/Taxonomy/Browser/wwwtax.cgi?mode=Info&id=985146&lvl=3&lin=f&keep=1&srchmode=1&unlock) | STIR04 3.3.2 | GCA_000223805.2 | NCBI | Stukenbrock et al. 2011 |
| [Zymoseptoria brevis](https://www.ncbi.nlm.nih.gov/Taxonomy/Browser/wwwtax.cgi?mode=Info&id=1047168&lvl=3&lin=f&keep=1&srchmode=1&unlock) | ZB163 | GCA_000983655.1 | NCBI | Grandaubert et al. 2015 |
| [Zymoseptoria brevis](https://www.ncbi.nlm.nih.gov/Taxonomy/Browser/wwwtax.cgi?mode=Info&id=1047168&lvl=3&lin=f&keep=1&srchmode=1&unlock) | Zb18110 | GCA_000966595.1 | NCBI | Grandaubert et al. 2015 |
| [Zymoseptoria passerinii](https://www.ncbi.nlm.nih.gov/Taxonomy/Browser/wwwtax.cgi?mode=Info&id=985148&lvl=3&lin=f&keep=1&srchmode=1&unlock) | SP63 | GCA_000223825.2 | NCBI | Stukenbrock et al. 2011 |
| [Zymoseptoria pseudotritici](https://www.ncbi.nlm.nih.gov/Taxonomy/Browser/wwwtax.cgi?mode=Info&id=1173715&lvl=3&lin=f&keep=1&srchmode=1&unlock) | ST04IR 5.5 | GCA_000983605.1 | NCBI | Grandaubert et al. 2015 |
| [Zymoseptoria pseudotritici](https://www.ncbi.nlm.nih.gov/Taxonomy/Browser/wwwtax.cgi?mode=Info&id=985140&lvl=3&lin=f&keep=1&srchmode=1&unlock) | STIR04 2.2.1 | GCA_000223665.2 | NCBI | Stukenbrock et al. 2011 |
| [Zymoseptoria pseudotritici](https://www.ncbi.nlm.nih.gov/Taxonomy/Browser/wwwtax.cgi?mode=Info&id=744267&lvl=3&lin=f&keep=1&srchmode=1&unlock) | STIR04 3.11.1 | GCA_000226675.2 | NCBI | Stukenbrock et al. 2011 |
| [Zymoseptoria pseudotritici](https://www.ncbi.nlm.nih.gov/Taxonomy/Browser/wwwtax.cgi?mode=Info&id=985143&lvl=3&lin=f&keep=1&srchmode=1&unlock) | STIR04 4.3.1 | GCA_000223685.2 | NCBI | Stukenbrock et al. 2011 |
| [Zymoseptoria pseudotritici](https://www.ncbi.nlm.nih.gov/Taxonomy/Browser/wwwtax.cgi?mode=Info&id=985141&lvl=3&lin=f&keep=1&srchmode=1&unlock) | STIR04 5.3 | GCA_000223705.2 | NCBI | Stukenbrock et al. 2011 |
| [Zymoseptoria pseudotritici](https://www.ncbi.nlm.nih.gov/Taxonomy/Browser/wwwtax.cgi?mode=Info&id=985142&lvl=3&lin=f&keep=1&srchmode=1&unlock) | STIR04 5.9.1 | GCA_000223725.2 | NCBI | Stukenbrock et al. 2011 |
| [Zymoseptoria tritici](https://www.ncbi.nlm.nih.gov/Taxonomy/Browser/wwwtax.cgi?mode=Info&id=1276529&lvl=3&lin=f&keep=1&srchmode=1&unlock) | ST99CH 1A5 | GCA_900099495.1 | NCBI | European Bioinformatics Institute (EBI) |
| [Zymoseptoria tritici](https://www.ncbi.nlm.nih.gov/Taxonomy/Browser/wwwtax.cgi?mode=Info&id=1276538&lvl=3&lin=f&keep=1&srchmode=1&unlock) | ST99CH 3D7 | GCA_900091695.1 | NCBI | European Bioinformatics Institute (EBI) |
| *Zymoseptoria tritici* | STIR04 A26b | GCA_000223645.2 | NCBI | Stukenbrock et al. 2011 |
| [Zymoseptoria tritici](https://www.ncbi.nlm.nih.gov/Taxonomy/Browser/wwwtax.cgi?mode=Info&id=985138&lvl=3&lin=f&keep=1&srchmode=1&unlock) | STIR04 A48b | GCA_000223625.2 | NCBI | Stukenbrock et al. 2011 |
