# Supplementary material for: Genome-scale data resolve ancestral rock-inhabiting lifestyle in Dothideomycetes (Ascomycota)
Source: IMA Fungus. 2019 Oct 30;10:19. doi: 10.1186/s43008-019-0018-2 (PMC7325674; doi:10.1186/s43008-019-0018-2)
Supplement: Supplementary file 2 — Additional file 2: Table S2. RF distances and normalized RF distance among the main phylogenies generated from the datasets containing the complete set of samples. Three distances are reported: a regular RF distance and two flavours of weighted RF distance as reported in the RAxML 8.2 manual. Numbers in the first two columns refer to the following starting datasets and reconstruction methods: (0) “> 1 kb Gblocks” IQTree run 1; (1) “> 1 kb Gblocks” IQTree run 2; (2) “> 1 kb Gblocks” IQTree run 3; (3) “> 1 kb Gblocks” ASTRAL; (4) “Complete Gblocks” IQTree; (5) “>1Kb GUIDANCE” IQTree; (6) “>1Kb GUIDANCE” ASTRAL. [file 43008_2019_18_MOESM2_ESM.docx]

**Table S2.** RF distances and normalized RF distance among the main phylogenies generated from the datasets containing the complete set of samples. Three distances are reported: a regular RF distance and two flavours of weighted RF distance as reported in RAxML 8.2 manual. Numbers in the first two columns refer to the following starting datasets and reconstruction methods: (0) “>1kb Gblocks” IQTree run 1, (1) “>1kb Gblocks” IQTree run 2, (2) “>1kb Gblocks” IQTree run 3, (3) “>1kb Gblocks” ASTRAL, (4) “Complete Gblocks” IQTree, (5) “>1Kb GUIDANCE” IQTree, (6) “>1Kb GUIDANCE” ASTRAL.

| **Phylogenies** | | **RF** | | **WRF1** | | **WRF2** | |
| --- | --- | --- | --- | --- | --- | --- | --- |
| 0 | 1 | 0 | 0,000 | 0,000 | 0,000 | 2,700 | 0,006 |
| 0 | 2 | 0 | 0,000 | 0,000 | 0,000 | 1,300 | 0,003 |
| 0 | 3 | 52 | 0,109 | 24,620 | 0,052 | 444,500 | 0,930 |
| 0 | 4 | 30 | 0,063 | 27,060 | 0,057 | 28,320 | 0,059 |
| 0 | 5 | 30 | 0,063 | 27,720 | 0,058 | 29,880 | 0,063 |
| 0 | 6 | 52 | 0,109 | 24,450 | 0,051 | 444,610 | 0,930 |
| 1 | 2 | 0 | 0,000 | 0,000 | 0,000 | 2,040 | 0,004 |
| 1 | 3 | 52 | 0,109 | 24,160 | 0,051 | 443,900 | 0,929 |
| 1 | 4 | 30 | 0,063 | 26,800 | 0,056 | 29,280 | 0,061 |
| 1 | 5 | 30 | 0,063 | 27,410 | 0,057 | 30,330 | 0,063 |
| 1 | 6 | 52 | 0,109 | 24,010 | 0,050 | 443,990 | 0,929 |
| 2 | 3 | 52 | 0,109 | 24,510 | 0,051 | 444,190 | 0,929 |
| 2 | 4 | 30 | 0,063 | 26,780 | 0,056 | 28,260 | 0,059 |
| 2 | 5 | 30 | 0,063 | 27,610 | 0,058 | 29,930 | 0,063 |
| 2 | 6 | 52 | 0,109 | 24,270 | 0,051 | 444,370 | 0,930 |
| 3 | 4 | 56 | 0,117 | 26,130 | 0,055 | 443,450 | 0,928 |
| 3 | 5 | 48 | 0,100 | 23,000 | 0,048 | 447,200 | 0,936 |
| 3 | 6 | 12 | 0,025 | 0,020 | 0,000 | 0,160 | 0,000 |
| 4 | 5 | 28 | 0,059 | 25,380 | 0,053 | 27,720 | 0,058 |
| 4 | 6 | 52 | 0,109 | 24,220 | 0,051 | 445,340 | 0,932 |
| 5 | 6 | 48 | 0,100 | 22,860 | 0,048 | 447,280 | 0,936 |
