# Supplementary material for: Genome-scale data resolve ancestral rock-inhabiting lifestyle in Dothideomycetes (Ascomycota)
Source: IMA Fungus. 2019 Oct 30;10:19. doi: 10.1186/s43008-019-0018-2 (PMC7325674; doi:10.1186/s43008-019-0018-2)
Supplement: Supplementary file 3 — Additional file 3: Table S3. RF distances and normalized RF distances from the reference topology of the 30 resampled matrices for each resampling effort value (0.1–30%). [file 43008_2019_18_MOESM3_ESM.docx]

**Table S3.** RF distances and normalized RF distances from the reference topology of the 30 resampled matrices for each resampling effort value (0.1-30 %).

|  | **0,1%** | | **1%** | | **10%** | | **20%** | | **30%** | |
| --- | --- | --- | --- | --- | --- | --- | --- | --- | --- | --- |
|  | 120 | 0,251 | 52 | 0,109 | 22 | 0,046 | 10 | 0,021 | 22 | 0,046 |
|  | 144 | 0,301 | 66 | 0,138 | 44 | 0,092 | 20 | 0,042 | 18 | 0,038 |
|  | 160 | 0,335 | 74 | 0,155 | 26 | 0,054 | 22 | 0,046 | 20 | 0,042 |
|  | 148 | 0,310 | 70 | 0,146 | 34 | 0,071 | 12 | 0,025 | 28 | 0,059 |
|  | 162 | 0,339 | 58 | 0,121 | 32 | 0,067 | 24 | 0,050 | 18 | 0,038 |
|  | 172 | 0,360 | 66 | 0,138 | 34 | 0,071 | 24 | 0,050 | 12 | 0,025 |
|  | 146 | 0,305 | 62 | 0,130 | 36 | 0,075 | 30 | 0,063 | 18 | 0,038 |
|  | 138 | 0,289 | 66 | 0,138 | 32 | 0,067 | 28 | 0,059 | 20 | 0,042 |
|  | 146 | 0,305 | 52 | 0,109 | 24 | 0,050 | 24 | 0,050 | 22 | 0,046 |
|  | 160 | 0,335 | 70 | 0,146 | 32 | 0,067 | 32 | 0,067 | 14 | 0,029 |
|  | 148 | 0,310 | 60 | 0,126 | 34 | 0,071 | 18 | 0,038 | 24 | 0,050 |
|  | 142 | 0,297 | 70 | 0,146 | 38 | 0,079 | 38 | 0,079 | 10 | 0,021 |
|  | 140 | 0,293 | 64 | 0,134 | 26 | 0,054 | 32 | 0,067 | 20 | 0,042 |
|  | 138 | 0,289 | 60 | 0,126 | 52 | 0,109 | 18 | 0,038 | 22 | 0,046 |
|  | 138 | 0,289 | 64 | 0,134 | 40 | 0,084 | 26 | 0,054 | 24 | 0,050 |
|  | 134 | 0,280 | 76 | 0,159 | 22 | 0,046 | 28 | 0,059 | 10 | 0,021 |
|  | 158 | 0,331 | 74 | 0,155 | 26 | 0,054 | 16 | 0,033 | 26 | 0,054 |
|  | 158 | 0,331 | 42 | 0,088 | 38 | 0,079 | 18 | 0,038 | 16 | 0,033 |
|  | 160 | 0,335 | 76 | 0,159 | 42 | 0,088 | 40 | 0,084 | 14 | 0,029 |
|  | 134 | 0,280 | 70 | 0,146 | 28 | 0,059 | 22 | 0,046 | 20 | 0,042 |
|  | 146 | 0,305 | 66 | 0,138 | 24 | 0,050 | 32 | 0,067 | 22 | 0,046 |
|  | 142 | 0,297 | 78 | 0,163 | 36 | 0,075 | 20 | 0,042 | 12 | 0,025 |
|  | 158 | 0,331 | 60 | 0,126 | 30 | 0,063 | 28 | 0,059 | 28 | 0,059 |
|  | 172 | 0,360 | 60 | 0,126 | 46 | 0,096 | 20 | 0,042 | 20 | 0,042 |
|  | 142 | 0,297 | 78 | 0,163 | 34 | 0,071 | 12 | 0,025 | 10 | 0,021 |
|  | 144 | 0,301 | 48 | 0,100 | 40 | 0,084 | 32 | 0,067 | 18 | 0,038 |
|  | 130 | 0,272 | 54 | 0,113 | 26 | 0,054 | 30 | 0,063 | 26 | 0,054 |
|  | 138 | 0,289 | 62 | 0,130 | 38 | 0,079 | 20 | 0,042 | 22 | 0,046 |
|  | 136 | 0,285 | 58 | 0,121 | 26 | 0,054 | 16 | 0,033 | 28 | 0,059 |
|  | 142 | 0,297 | 62 | 0,130 | 26 | 0,054 | 26 | 0,054 | 24 | 0,050 |
| **Average** | 147 | 0,307 | 64 | 0,134 | 33 | 0,069 | 24 | 0,050 | 20 | 0,041 |
| **SD** | 12 | 0,026 | 9 | 0,019 | 8 | 0,016 | 7 | 0,016 | 5 | 0,011 |
