# Supplementary material for: Genome-scale data resolve ancestral rock-inhabiting lifestyle in Dothideomycetes (Ascomycota)
Source: IMA Fungus. 2019 Oct 30;10:19. doi: 10.1186/s43008-019-0018-2 (PMC7325674; doi:10.1186/s43008-019-0018-2)
Supplement: Supplementary file 4 — Additional file 4: Table S4. Average values and standard deviation (SD) of RF distances and normalized RF distances among the 30 phylogenies generated from the randomly resampled matrix with the same resampling effort. [file 43008_2019_18_MOESM4_ESM.docx]

**Table S4.** Average values and standard deviation (SD) of RF distances and normalized RF distances among the 30 phylogenies generated from the randomly resampled matrix with the same resampling effort.

|  | **0,1%** | | **1%** | | **10%** | | **20%** | | **30%** | |
| --- | --- | --- | --- | --- | --- | --- | --- | --- | --- | --- |
|  | **RF** | **nRF** | **RF** | **nRF** | **RF** | **nRF** | **RF** | **nRF** | **RF** | **nRF** |
| **Average** | 183 | 0,384 | 75 | 0,158 | 41 | 0,086 | 32 | 0,067 | 26 | 0,055 |
| **SD** | 19 | 0,036 | 10 | 0,019 | 8 | 0,016 | 8 | 0,016 | 7 | 0,014 |
