# Supplementary material for: Genome-scale data resolve ancestral rock-inhabiting lifestyle in Dothideomycetes (Ascomycota)
Source: IMA Fungus. 2019 Oct 30;10:19. doi: 10.1186/s43008-019-0018-2 (PMC7325674; doi:10.1186/s43008-019-0018-2)
Supplement: Supplementary file 5 — Additional file 5: Table S5. Taxa and associated lifestyle used in ancestral character state reconstruction (lichen: 0; rock-inhabiting: 1; plant pathogen: 2; saprotroph: 3; epiphyte: 4; animal pathogen: 5; fungi pathogen: 6; ectomycorrhiza: 7; endophyte: 8; extremophile: 9). [file 43008_2019_18_MOESM5_ESM.docx]

**Table S5.** Taxa and associated lifestyle used in ancestral character state reconstruction (lichen: 0; rock-inhabiting: 1; plant pathogen: 2; saprotroph: 3; epiphyte: 4; animal pathogen: 5; fungi pathogen: 6; ectomycorrhiza: 7; endophyte: 8; extremophile: 9).

| Trypethelium eluteriae JGI 1006394 | 0 | Baudoinia panamericana UAMH 10762 | 3 | Zymoseptoria tritici ST99CH 3D7 | 2 | Cercospora sojina S9 | 2 | Dothistroma septosporum NZE10 v1.0 | 2 | Phyllosticta capitalensis Gm33 | 2 | Macrophomina phaseolina MP00065 | 2 | Nigrograna mackinnonii E5202H | 5 | Macroventuria anomochaeta CBS 525.71 | 3 | Phoma tracheiphila IPT5 | 2 | Curvularia papendorfii UM 226 | 2 |
| --- | --- | --- | --- | --- | --- | --- | --- | --- | --- | --- | --- | --- | --- | --- | --- | --- | --- | --- | --- | --- | --- |
| Cryomyces antarcticus CCFEE 534 | 1 | Hortaea werneckii EXF 2000M0 scaffolds | 9 | Zymoseptoria brevis ZB163 | 2 | Lecanosticta acicola CBS 871.95 | 2 | Dothistroma septosporum MU NZE8 | 2 | Phyllosticta citribraziliensis CBS 100098 | 2 | Macrophomina phaseolina MP00325 | 2 | Lophiostoma macrostomum 1011357 | 3 | Didymella exigua CBS 183.55 | 3 | Leptosphaeria maculans JN3 | 2 | Bipolaris oryzae ATCC 44560 | 2 |
| Elsinoe ampelina CECT 20119 | 2 | Hortaea werneckii EXF 2000 | 9 | Zymoseptoria brevis Zb18110 | 2 | Zasmidium cellare ATCC 36951 | 3 | Dothistroma septosporum NZFS4520 | 2 | Phyllosticta citrichinaensis CBS 130529 | 2 | Macrophomina phaseolina MP00327 | 2 | Sporormia fimetaria 1011321 | 3 | Didymella zeae maydis 3018 | 2 | Leptosphaeria maculans Lepmu1 | 2 | Bipolaris oryzae TG12bL2 | 2 |
| Myriangium duriaei CBS 260.36 | 5 | Teratosphaeria nubilosa CBS 116005 | 2 | Zymoseptoria pseudotritici STIR04 4.3.1 | 2 | Mycosphaerella laricina CBS 326.52 | 2 | Lineolata rhizophorae ATCC16933 | 4 | Phyllosticta citriasiana 1011301 | 2 | Aliquandostipite khaoyaiensis CBS 118232 | 3 | Westerdykella ornata CBS 379.55 | 3 | Phoma herbarum JCM 15942 | 2 | Clathrospora elynae CBS 161.51 | 4 | Bipolaris victoriae FI3 | 2 |
| Delphinella strobiligena CBS 735.71 | 3 | Piedraia hortae 1011305 | 5 | Zymoseptoria pseudotritici STIR04 5.9.1 | 2 | Cladosporium fulvum | 2 | Aulographum hederae 1006065 | 3 | Phyllosticta citricarpa CGMCC3.14348 | 2 | Patellaria atrata CBS 101060 | 3 | Melanomma pulvis pyrius 1011365 | 3 | Stagonosporopsis tanaceti CBS 131484 | 2 | Decorospora gaudefroyi 1032355 | 3 | Bipolaris zeicola 26 R 13 | 2 |
| Aureobasidium pullulans SAMN04565625 | 3 | Acidomyces richmondensis BFW | 9 | Zymoseptoria pseudotritici ST04IR 5.5 | 2 | Mycosphaerella arachidis CALF 13A | 2 | Rhizodiscina lignyota CBS 133067 | 3 | Phyllosticta citricarpa Gc12 | 2 | Pseudovirgaria hyperparasitica CBS 121739 | 6 | Beverwykella pulmonaria JCM 9230 | 3 | Epicoccum nigrum ICMP 19927 | 3 | Alternaria consortialis JCM 1940 | 2 | Bipolaris sorokiniana ND90Pr | 2 |
| Aureobasidium pullulans var. pullulans EXF 150 | 3 | Acidomyces richmondensis meta | 9 | Zymoseptoria pseudotritici STIR04 5.3 | 2 | Dothistroma pini CBS 116487 | 2 | Eremomyces bilateralis CBS 781.70 | 3 | Phyllosticta sp. CPC 27169 | 2 | Lophium mytilinum CBS 269.34 | 4 | Pleomassaria siparia 1011309 | 3 | Epicoccum sorghinum USPMTOX48 | 2 | Alternaria brassicicola ATCC 96836 | 2 | Bipolaris maydis ATCC 48331 | 2 |
| Aureobasidium sp. FSWF8 4 | 3 | Dissoconium aciculare 1011337 | 6 | Zymoseptoria pseudotritici STIR04 2.2.1 | 2 | Dothistroma septosporum CMW 37193 | 2 | Microthyrium microscopicum CBS 115976 | 3 | Phyllosticta sp. CPC 27913 | 2 | Mytilinidion resinicola CBS 304.34 | 4 | Massariosphaeria phaeospora CBS 611.86 | ? | Dothidotthia symphoricarpi 1011345 | 2 | Alternaria arborescens EGS 39 128 | 2 | Cochliobolus heterostrophus C5 1 | 2 |
| Aureobasidium pullulans var. subglaciale EXF 2481 | 3 | Ramularia collo cygni DK05 | 2 | Zymoseptoria pseudotritici STIR04 3.11.1 | 2 | Dothistroma septosporum CMW 37194 | 2 | Trichodelitschia bisporula CBS 262.69 | 3 | Lasiodiplodia theobromae CSS 01s | 2 | Lepidopterella palustris CBS 459.81 | 3 | Corynespora cassiicola UM 591 | 2 | Shiraia sp. slf14 | 8 | Alternaria alternata B2a | 2 | Bipolaris maydis C5 | 2 |
| Aureobasidium pullulans var. namibiae CBS 147.97 | 3 | Ramularia endophylla CBS 113265 | 2 | Phaeocryptopus gaeumannii CBS 267.37 | 2 | Dothistroma septosporum CMW 38941 | 2 | Ochroconis constricta UM 578 | 5 | Diplodia corticola CBS 112549 | 2 | Cenococcum geophilum 1.58 | 7 | Corynespora cassiicola CCP | 2 | Paraphoma sp. B47 9 | ? | Alternaria alternata SRC1lrK2f | 2 | Cochliobolus heterostrophus C5 3 | 2 |
| Aureobasidium melanogenum HN6.2 | 3 | Zymoseptoria passerinii SP63 | 2 | Pseudocercospora fijiensis CIRAD86 | 2 | Dothistroma septosporum CMW 44207 | 2 | Verruconis gallopava CBS 43764 | 5 | Diplodia seriata DS831 | 2 | Glonium stellatum CBS 207.34 | 3 | Corynespora cassiicola SAMEA103891068 | 2 | Setomelanomma holmii CBS 110217 | 2 | Alternaria alternata Z7 | 2 | L1844 | 1 |
| Aureobasidium pullulans AY4 | 3 | Zymoseptoria ardabiliae STIR04 1.1.1 | 2 | Pseudocercospora pini densiflorae CBS 125139 | 2 | Dothistroma septosporum CMW 14822 | 2 | Tothia fuscella CBS 130266 | ? | Diplodia seriata F98.1 | 2 | Hysterium pulicare CBS 123377 | 3 | Trematosphaeria pertusa CBS 122368 | 3 | Ophiobolus disseminans CBS 113818 | 3 | Alternaria alternata 133aPRJ | 2 | L1853 | 1 |
| Aureobasidium pullulans var. melanogenum CBS 110374 | 3 | Zymoseptoria ardabiliae STIR04 1.1.2 | 2 | Mycosphaerella eumusae CBS 114824 | 2 | Dothistroma septosporum CMW 14823 | 2 | Venturia carpophila JP3 5 | 2 | Diplodia scrobiculata CMW30223 | 2 | Rhytidhysteron rufulum CBS 306.38 | 3 | Bimuria novae zelandiae CBS 107.79 | 3 | Phaeosphaeriaceae sp. PMI 808 | 8 | Alternaria alternata ATCC 34957 | 2 | Saxomyces 5470 | 1 |
| Leptoxyphium fumago SC3815 | ? | Zymoseptoria ardabiliae ST11IR 6.1.1 | 2 | Pseudocercospora musae CBS 116634 | 2 | Dothistroma septosporum CMW 23429 | 2 | Venturia effusa 3Des10b | 2 | Diplodia sapinea CMW39103 | 2 | Delitschia confertaspora ATCC 74209 | 3 | Didymocrea sadasivanii CBS 438.65 | 3 | Stagonospora nodorum SN15 | 2 | Pyrenophora seminiperda CCB06 | 2 | L2282 | 1 |
| Polychaeton citri 1011313 | 4 | Zymoseptoria ardabiliae STIR04 3.13.1 | 2 | Mycosphaerella sp. Ston1 | 2 | Dothistroma septosporum CMW 37965 | 2 | Venturia pyrina ICMP 11032 | 2 | Diplodia sapinea CMW 190 | 2 | Zopfia rhizophila 1011325 | 2 | Karstenula rhodostoma CBS 690.94 | 3 | Phaeosphaeria nodorum Sn79 1.0 | 2 | Pyrenophora teres f. teres 0 1 | 2 | Curvularia papendorfii UM 226 | 2 |
| Peltaster fructicola LNHT1506 | 2 | Zymoseptoria ardabiliae STIR04 3.3.2 | 2 | Sphaerulina populicola P02.02b | 2 | Dothistroma septosporum CMW 13123 | 2 | Venturia inaequalis 1389 | 2 | Neofusicoccum parvum UCRNP2 | 2 | Clohesyomyces aquaticus CBS 115471 | 3 | Paraphaeosphaeria sporulosa AP3s5 JAC2a | 3 | Ampelomyces quisqualis HMLAC05119 | 6 | Pyrenophora tritici repentis Pt 1C BFP | 2 | Bipolaris oryzae ATCC 44560 | 2 |
| Rachicladosporium sp. CCFEE 5018 | 1 | Zymoseptoria tritici STIR04 A26b | 2 | Mycosphaerella sp. PB 2012b Mex 2 1 2 | 2 | Dothistroma septosporum CMW 40004 | 2 | Venturia inaequalis 1639 | 2 | Botryosphaeria dothidea Botdo1 1 | 2 | Lindgomyces ingoldianus ATCC 200398 | ? | Lentithecium fluviatile 1006093 | 3 | Stagonospora sp. SRC1lsM3a | ? | Stemphylium lycopersici CIDEFI 216 | 2 | Bipolaris oryzae TG12bL2 | 2 |
| Rachicladosporium antarcticum CCFEE 5527 | 1 | Zymoseptoria tritici STIR04 A48b | 2 | Sphaerulina musiva SO2202 | 2 | Dothistroma septosporum CMW 13121 | 2 | Venturia inaequalis EU B04 | 2 | Botryosphaeria dothidea LW030101 | 2 | Amniculicola lignicola CBS 123094 | 3 | Periconia macrospinosa DSE2036 | 8 | Pleosporales sp. UM 1110 | ? | Setosphaeria turcica Et28A | 2 | Bipolaris victoriae FI3 | 2 |
| Preussia sp. BSL10 | 3 | Mycosphaerella graminicola 16205 | 2 | Cercospora canescens BHU | 2 | Dothistroma septosporum CMW 44656 | 2 | Venturia inaequalis ICMP 13258 | 2 | Macrophomina phaseolina MRf1 | 2 | Verruculina enalia CBS 304.66 | 3 | Massarina eburnea CBS 473.64 | 3 | Pyrenochaeta sp. DS3sAY3a | ? | Setosphaeria turcica NY001 | 2 | Bipolaris zeicola 26 R 13 | 2 |
| Cladosporium sphaerospermum UM 843 | 3 | Zymoseptoria tritici ST99CH 1A5 | 2 | Cercospora zeae maydis 401984 | 2 | Dothistroma septosporum CMW 11305 | 2 | Coniosporium apollinis CBS 100218 | 1 | Macrophomina phaseolina MS6 | 2 | Lophiotrema nucula CBS 627.86 | ? | Byssothecium circinans CBS 675.92 | 3 | Pyrenochaeta lycopersici CRA PAV ER 1211 | 2 | Curvularia sp. IFB Z10 | ? | Bipolaris sorokiniana ND90Pr | 2 |
| Cladosporium sphaerospermum IMV 00045 | 3 | Baudoinia panamericana UAMH 10762 | 3 | Cercospora cf. sigesbeckiae PP 2012 071 | 2 | Dothistroma septosporum CMW 15843 | 2 | Saccharata proteae CBS 121410 | 2 | Macrophomina phaseolina MO00014 | 2 | Polyplosphaeria fusca CBS 125425 | 2 | Helminthosporium solani B AC 16A | 2 | Cucurbitaria berberidis CBS 394.84 | 3 | Cochliobolus lunatus m118 | 2 | Bipolaris maydis ATCC 48331 | 2 |
| Hortaea acidophila CBS 113389 | 9 | Hortaea werneckii EXF 2000M0 scaffolds | 9 | Cercospora sojina FLS21 | 2 | Dothistroma septosporum CMW 10211 | 2 | Aplosporella prunicola CBS 121.167 | 3 | Macrophomina phaseolina MP00003 | 2 | Aaosphaeria arxii CBS 175.79 | ? | Ascochyta rabiei ArDII | 2 | Pyrenochaeta sp. UM 256 | ? | Curvularia lunata CX 3 | 2 | Cochliobolus heterostrophus C5 1 | 2 |
