# Supplementary material for: Genome-scale data resolve ancestral rock-inhabiting lifestyle in Dothideomycetes (Ascomycota)
Source: IMA Fungus. 2019 Oct 30;10:19. doi: 10.1186/s43008-019-0018-2 (PMC7325674; doi:10.1186/s43008-019-0018-2)
Supplement: Supplementary file 6 — Additional file 6: Figure S1. Assembly completeness on the base of 3156 Pezizomycotina orthologs evaluated by BUSCO and expressed as the percentage of complete (green), duplicated (pink) and fragmented or missing (grey) genes. Distribution outliers are highlighted with “*”. [file 43008_2019_18_MOESM6_ESM.pdf]

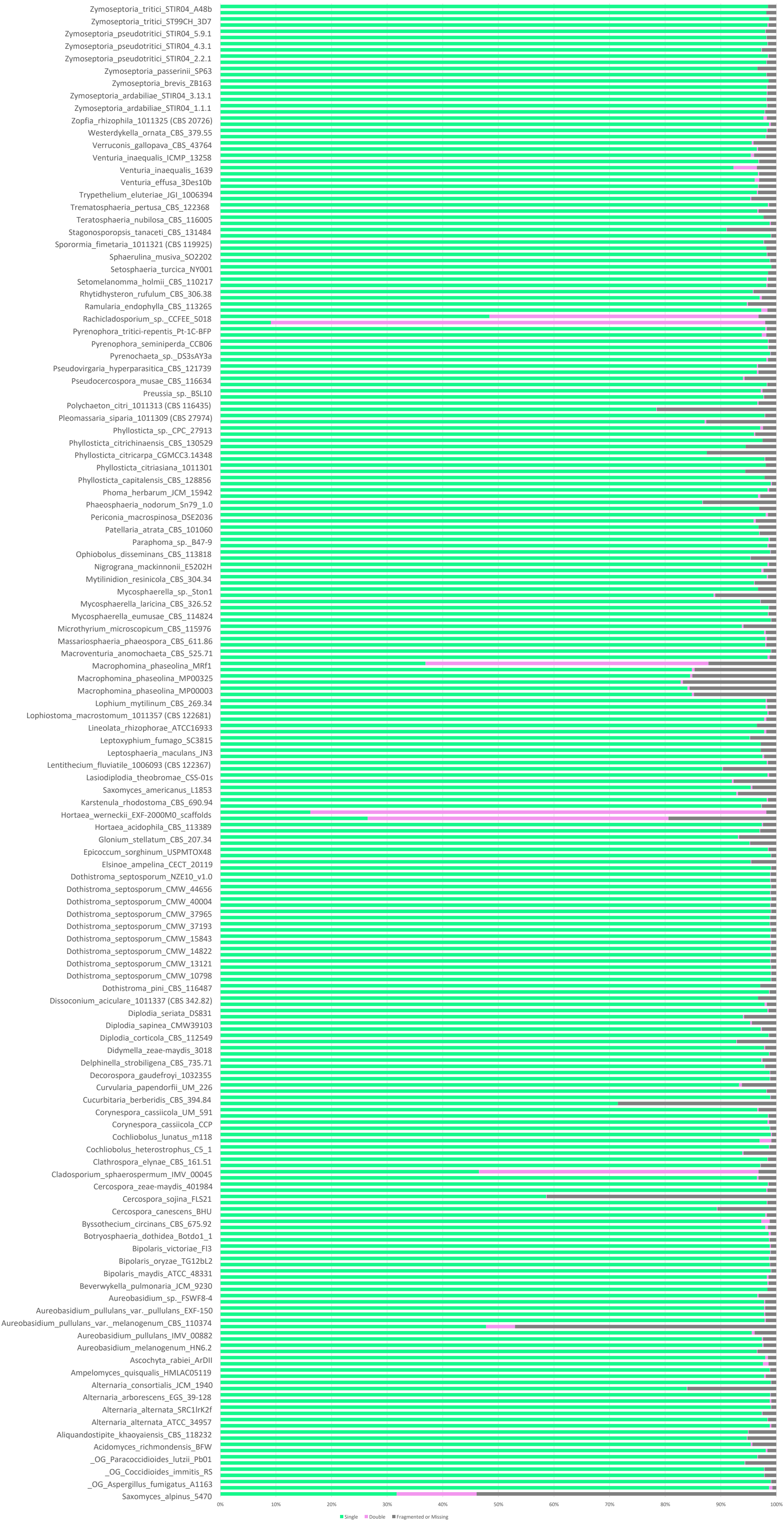

Figure S1. Assembly completeness on the base of 3156 Pezizomycotina orthologs evaluated by BUSCO and expressed as the percentage of complete (green), duplicated (pink) and fragmented or missing (grey) genes. Distribution outliers are highlighted with "*".
