# Supplementary figures and images for: Genome-scale data resolve ancestral rock-inhabiting lifestyle in Dothideomycetes (Ascomycota)
Source: IMA Fungus. 2019 Oct 30;10:19. doi: 10.1186/s43008-019-0018-2 (PMC7325674; doi:10.1186/s43008-019-0018-2)

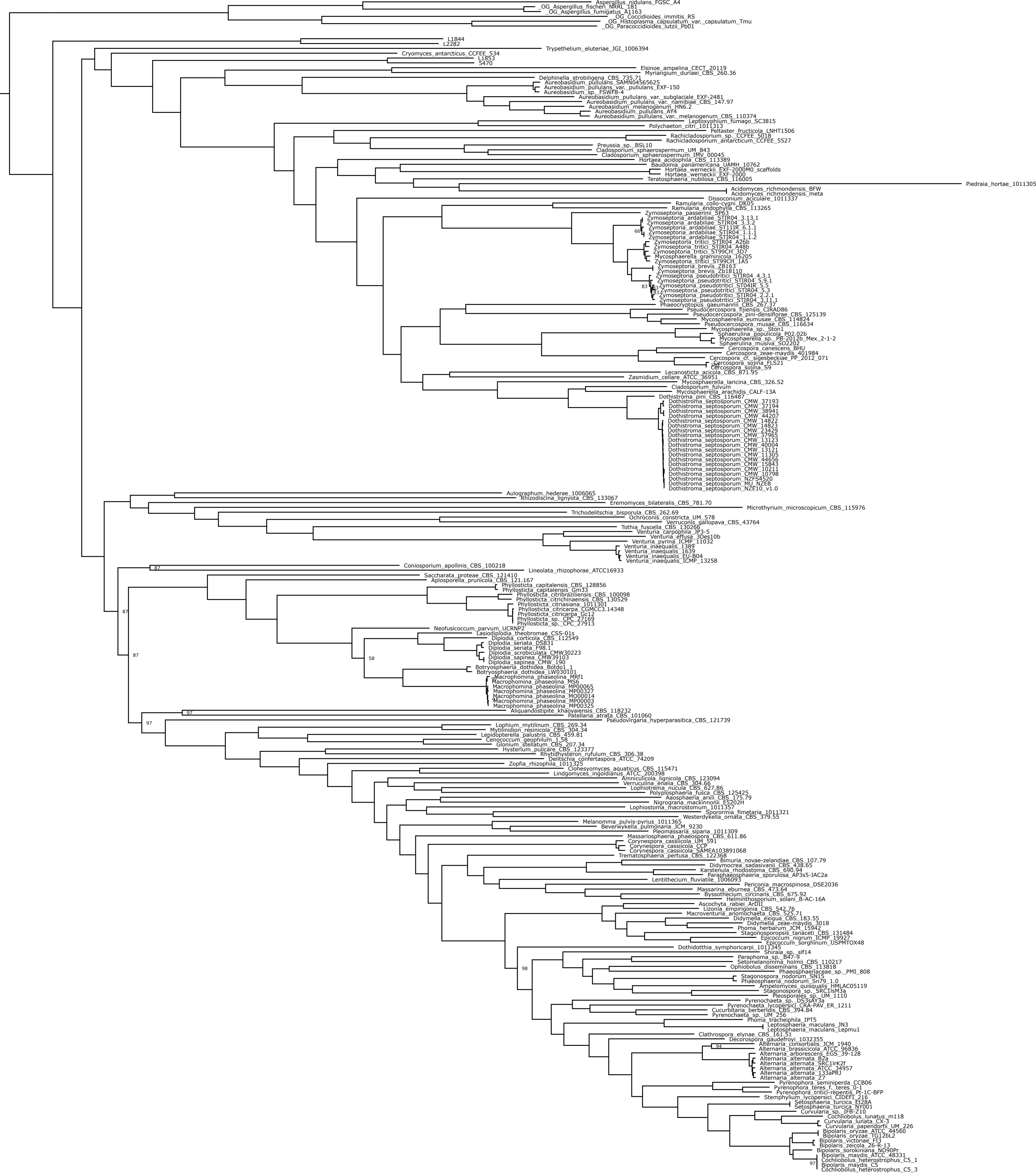

Supplement: Supplementary file 7 — Additional file 7: Figure S2. Phylogeny generated from 2998 concatenated genes of the “Complete Gblocks” dataset with IQTree. [file 43008_2019_18_MOESM7_ESM.pdf]

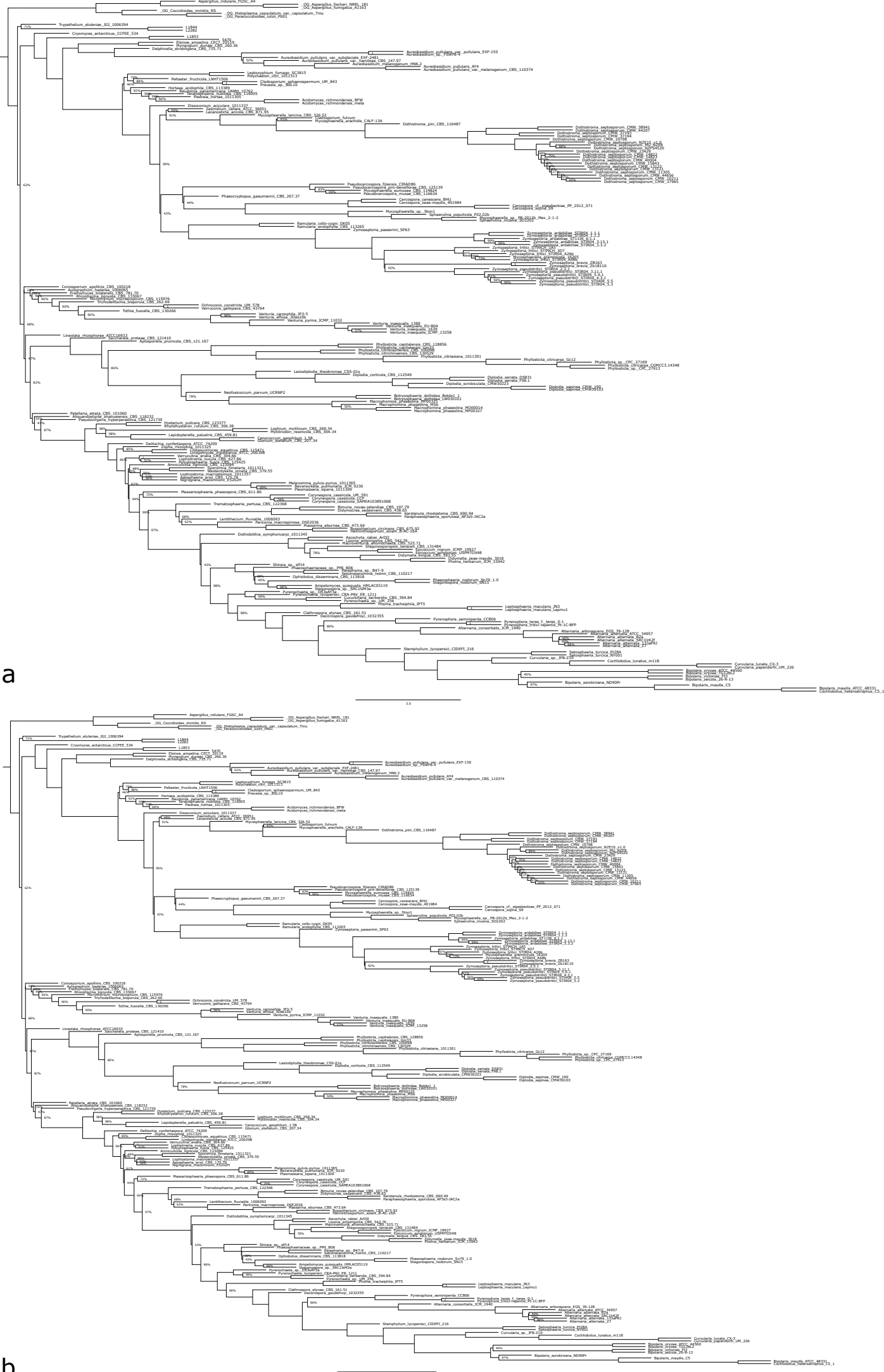

Supplement: Supplementary file 8 — Additional file 8: Figure S3. Phylogenies generated from 63 genes of the “No missing” dataset; (a) ML tree generated with IQTree on concatenated genes; (b) ASTRAL III species tree. [file 43008_2019_18_MOESM8_ESM.pdf]
