# Supplementary material for: Genome-scale data resolve ancestral rock-inhabiting lifestyle in Dothideomycetes (Ascomycota)
Source: IMA Fungus. 2019 Oct 30;10:19. doi: 10.1186/s43008-019-0018-2 (PMC7325674; doi:10.1186/s43008-019-0018-2)
Supplement: Supplementary file 9 — Additional file 9: Figure S4. Phylogeny generated from 1260 concatenated genes of the “>1Kb GUIDANCE” dataset with IQTree. [file 43008_2019_18_MOESM9_ESM.pdf]

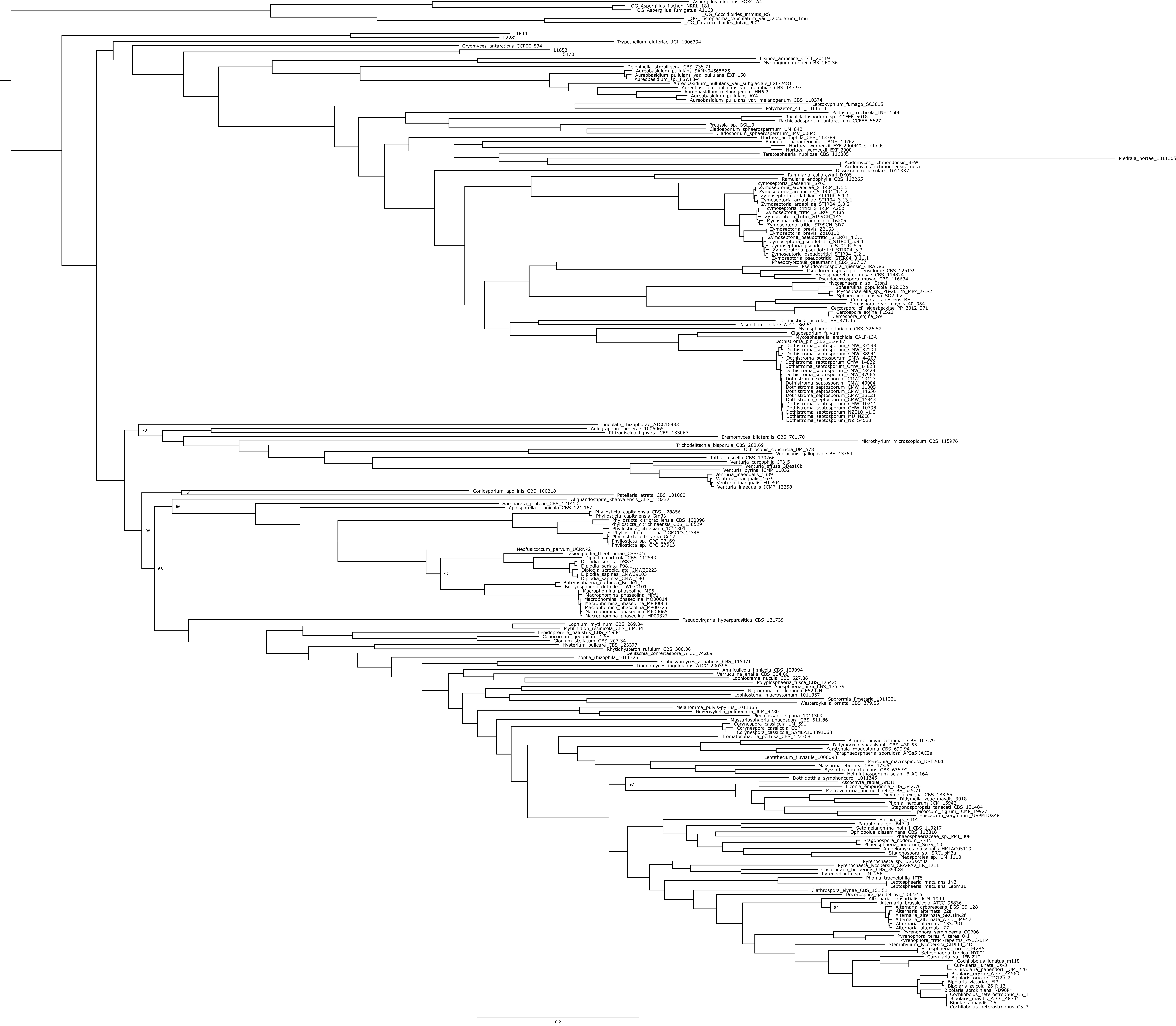

Figure S4. 1260 concatenated genes phylogeny generated from ">1Kb GUIDANCE" dataset with IQTree. Support values different from 100 are shown.
