# Supplementary material for: Genome-scale data resolve ancestral rock-inhabiting lifestyle in Dothideomycetes (Ascomycota)
Source: IMA Fungus. 2019 Oct 30;10:19. doi: 10.1186/s43008-019-0018-2 (PMC7325674; doi:10.1186/s43008-019-0018-2)
Supplement: Supplementary file 10 — Additional file 10: Figure S5. Phylogeny generated from 1260 concatenated genes of the “>1Kb GUIDANCE” dataset with ASTRAL. [file 43008_2019_18_MOESM10_ESM.pdf]

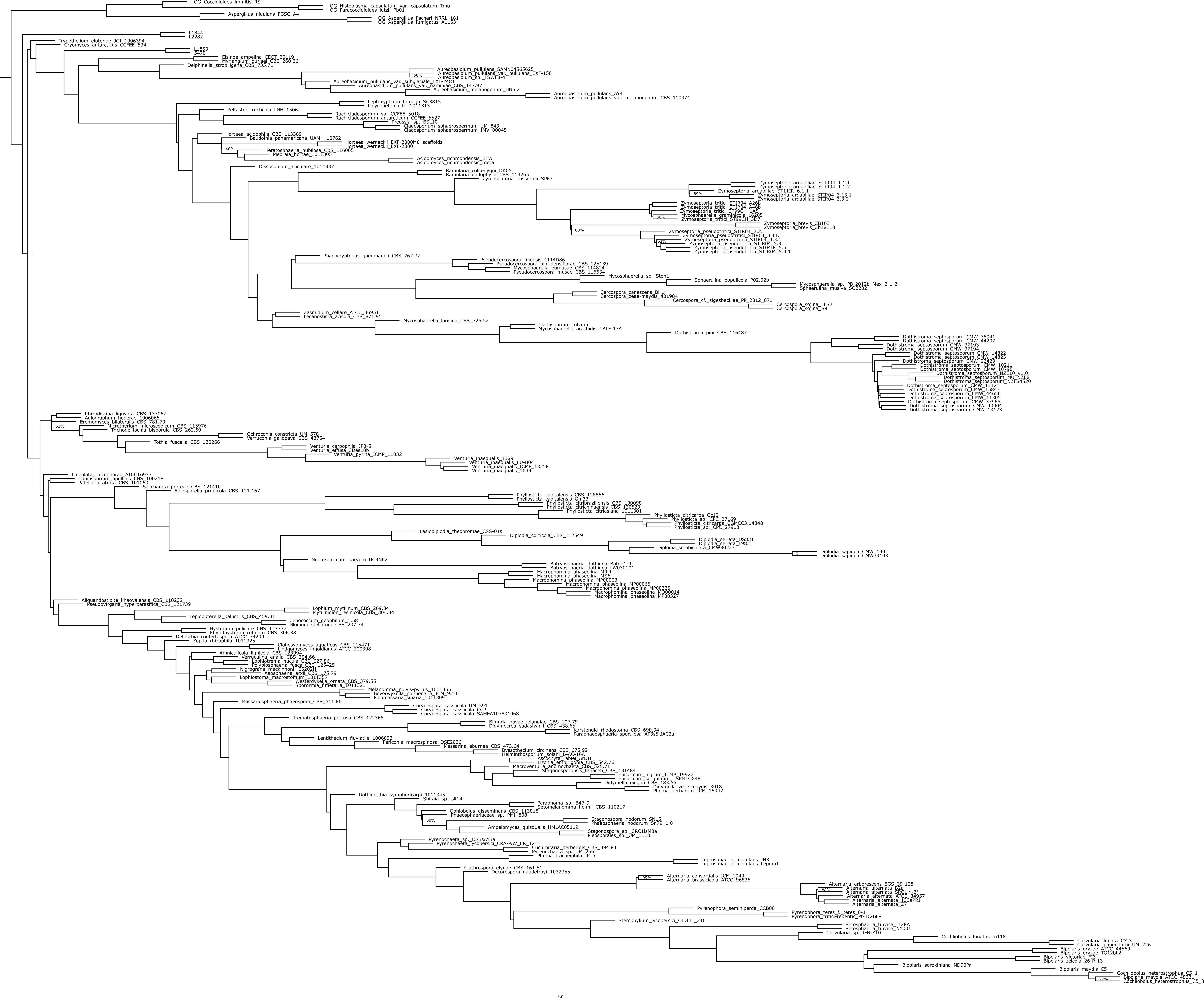

Figure S5. 1260 concatenated genes phylogeny generated from ">1Kb GUIDANCE" dataset with ASTRAL. Support values different from 100 are shown.
